# High-resolution EPR distance measurements on RNA and DNA with the non-covalent Ǵ spin label

**DOI:** 10.1093/nar/gkz1096

**Published:** 2019-11-28

**Authors:** Marcel Heinz, Nicole Erlenbach, Lukas S Stelzl, Grace Thierolf, Nilesh R Kamble, Snorri Th Sigurdsson, Thomas F Prisner, Gerhard Hummer

**Affiliations:** 1 Department of Theoretical Biophysics, Max Planck Institute of Biophysics, Max-von-Laue-Straße 3, 60438 Frankfurt am Main, Germany; 2 Institute of Physical and Theoretical Chemistry and Center of Biomolecular Magnetic Resonance, Goethe University Frankfurt, Max-von-Laue-Straße 7, 60438 Frankfurt am Main, Germany; 3 Department of Chemistry, Science Institute, University of Iceland, Dunhaga 3, 107 Reykjavk, Iceland; 4 Institute for Biophysics, Goethe University Frankfurt, 60438 Frankfurt am Main, Germany

## Abstract

Pulsed electron paramagnetic resonance (EPR) experiments, among them most prominently pulsed electron-electron double resonance experiments (PELDOR/DEER), resolve the conformational dynamics of nucleic acids with high resolution. The wide application of these powerful experiments is limited by the synthetic complexity of some of the best-performing spin labels. The recently developed $\bf\acute{G}$ (G-spin) label, an isoindoline-nitroxide derivative of guanine, can be incorporated non-covalently into DNA and RNA duplexes via Watson-Crick base pairing in an abasic site. We used PELDOR and molecular dynamics (MD) simulations to characterize $\bf\acute{G}$, obtaining excellent agreement between experiments and time traces calculated from MD simulations of RNA and DNA double helices with explicitly modeled $\bf\acute{G}$ bound in two abasic sites. The MD simulations reveal stable hydrogen bonds between the spin labels and the paired cytosines. The abasic sites do not significantly perturb the helical structure. $\bf\acute{G}$ remains rigidly bound to helical RNA and DNA. The distance distributions between the two bound $\bf\acute{G}$ labels are not substantially broadened by spin-label motions in the abasic site and agree well between experiment and MD. $\bf\acute{G}$ and similar non-covalently attached spin labels promise high-quality distance and orientation information, also of complexes of nucleic acids and proteins.

## INTRODUCTION

Pulsed EPR experiments can be used to probe the global structure and flexibility of nucleic acids with high resolution (1). In particular, pulsed electron-electron double resonance experiments (2) (PELDOR, also referred to as DEER) complement the structure determination by X-ray crystallography (3), nuclear magnetic resonance (4–6) (NMR) and cryo-EM experiments (7–9). PELDOR provides detailed information on distances even in highly dynamic systems, where traditional structure determination is not possible, reporting on the conformational flexibility of proteins, nucleic acids and protein-nucleic acid complexes (10,11). In rigid systems, PELDOR can even provide angular information (12). Typically, PELDOR experiments require the introduction of a pair of spin labels. The nature of the spin label is a critical issue. Flexible spin labels complicate the determination of high-resolution distances (13) and do not permit the extraction of angular information. By contrast, the rigid and covalently attached spin label **Ç** (C-spin) (14,15) enables highly accurate distance and angle measurements on, e.g. DNA. However, the synthesis of the **Ç** itself and especially of **Ç**-labeled nucleic acids remains difficult, limiting the wide applicability of high-resolution pulsed EPR experiments on nucleic acids.

A number of different spin labels were developed in the last years (16–19), but there is still a lack of spin labels that can be employed without significant synthetic effort. One way to reduce the synthetic effort, which is particularly relevant for larger nucleic acids of biological interest, is to incorporate the spin label non-covalently. There, the challenge is to achieve high-affinity and high specificity binding to the nucleic acid target molecule (20–22). Note that specific non-covalent incorporation of **Ç** derivatives in nucleic acids, albeit a promising strategy, has not been achieved (23). Recently we showed that the $\bf\acute{G}$ (G-spin) incorporates with high affinity and specificity into abasic sites in double-stranded RNA (dsRNA) and double-stranded DNA (dsDNA) (24). Furthermore, first measurements of labeled dsRNA revealed orientation selection in the measured PELDOR time traces, which permits the extraction of angular information.

Here we use molecular dynamics (MD) simulations to study the impact of $\bf\acute{G}$ incorporation on nucleic acid structure and conformational dynamics. It has been shown that native DNA (25), as well as larger photolabile protecting groups covalently attached at DNA bases (26), can be well described by state-of-the-art MD simulations. Despite the fact that current RNA force fields do not fully reflect experimental observations for single-stranded RNAs (27), native and protonated bases in double-stranded RNAs are described well (28–31). For dsRNA with TEMPO-labeled nucleotides, MD simulations have produced distance distributions in very promising agreement with PELDOR measurements (32).

With the methods on hand to calculate the PELDOR time traces from MD simulation (1,33) and to explicitly model the spin label in the MD simulations of DNA and RNA for a direct comparison with PELDOR experiments, we elucidate the influence of the non-covalently attached $\bf\acute{G}$ on dsRNA and dsDNA structure and motions. Conversely, we validate state-of-the-art nucleic acid force fields for DNA and RNA against PELDOR experiments.

## MATERIALS AND METHODS

### Preparation of $\bf\acute{G}$-labeled oligonucleotide duplexes for PELDOR measurements

The oligonucleotide duplexes containing two abasic sites were noncovalently spin-labeled by admixing stock solutions of the spin label $\bf\acute{G}$ (20 nmol) and the different nucleic acids (10 nmol). The solvent was removed *in vacuo* and the residue dissolved in a phosphate buffer (100 μl; 10 mM NaHPO_4_, 100 mM NaCl, 0.1 mM Na_2_EDTA, pH 7.0) and annealed: 90°C for 2 min, 60°C for 5 min, 50°C for 5 min and 22°C for 15 min. The water was subsequently removed *in vacuo* and the residue dissolved in an aqueous 30% ethylene glycol solution (100 μl).

RNA sequence, where “_” denotes the abasic site:

5′-CGAG-_AU-CGC-GCG-CGA-UCC-UCG-3′

3′-GCUC-CUA-GCG-CGC-GCU-A_G-AGC-5′

DNA sequence, where “_” denotes the abasic site:

5′-T-GT-CA_-TCG-CGC-GCG-CGC-ATC-3′

3′-CA-GTC-AGC-GC_-CGC-GCG-TAG-T-5′

### X-band measurements

A Bruker Elexsys E580 X/Q-band spectrometer equipped with an Oxford CF935 cryostat was used with a Bruker MS3 3mm loop gap resonator for the X-band measurements (0.3 T/9 GHz). Microwave pulses were amplified by a 1 kW Travelling Wave Tube (TWT). 32 ns ($\frac{\pi }{2}$ and π) pulses were used for detection and a 20 ns (π) pump pulse. The delay between the first two probe pulses was 132 ns. The separation to the second π-probe pulse was 1.8 μs. The repetition time of the experiment was 6 ms. The frequency of the pump pulse was fixed to the intensity maximum of the nitroxide powder spectrum to obtain maximum pumping efficiency. The probe frequency was chosen to be 40/55/70/85 MHz (DNA) and 40/50/60/75/90 MHz (RNA) above this frequency. All experiments were performed at 50 K using a continuous flow of liquid helium and the temperature maintained using an Oxford Instruments ITC 503 temperature control unit.

### G-band measurements

All G-band EPR experiments (180 GHz/6.4 T) were performed with an in-house built G-band spectrometer equipped with two independent frequency sources (34). The probe pulse lengths were between 32–40 ns for the $\frac{\pi }{2}$-pulse, 60–70 ns for the π-pulse. The pump inversion pulse was 30–38 ns. The pulse separation between the first probe pulses was 220 ns and 1.8 μs to the next probe π-pulse. The repetition time of the experiment was 6 ms. All experiments were carried out at a temperature of 40 K. Every set of experiments consisted of ∼40–120 time traces averaged with 100 shots per point and were recorded at different field positions across the EPR spectrum, corresponding roughly to the *B*∥*g*_*xx*_, *B*∥*g*_*yy*_, *B*∥*g*_*zz*_ and two additional positions in between. The probe frequency was set in all cases at a constant offset of 60 MHz above (DNA)/below (RNA) the pump frequency.

### Experimental PELDOR data processing

The intramolecular PELDOR form factor was extracted from the raw data using the DeerAnalysis2018 software package (35) with homogenous 3D background correction. The experimental PELDOR time traces before and after background correction are available in the Supporting Informations (Supplementary Figures S1 and S2).

**Figure 1.**
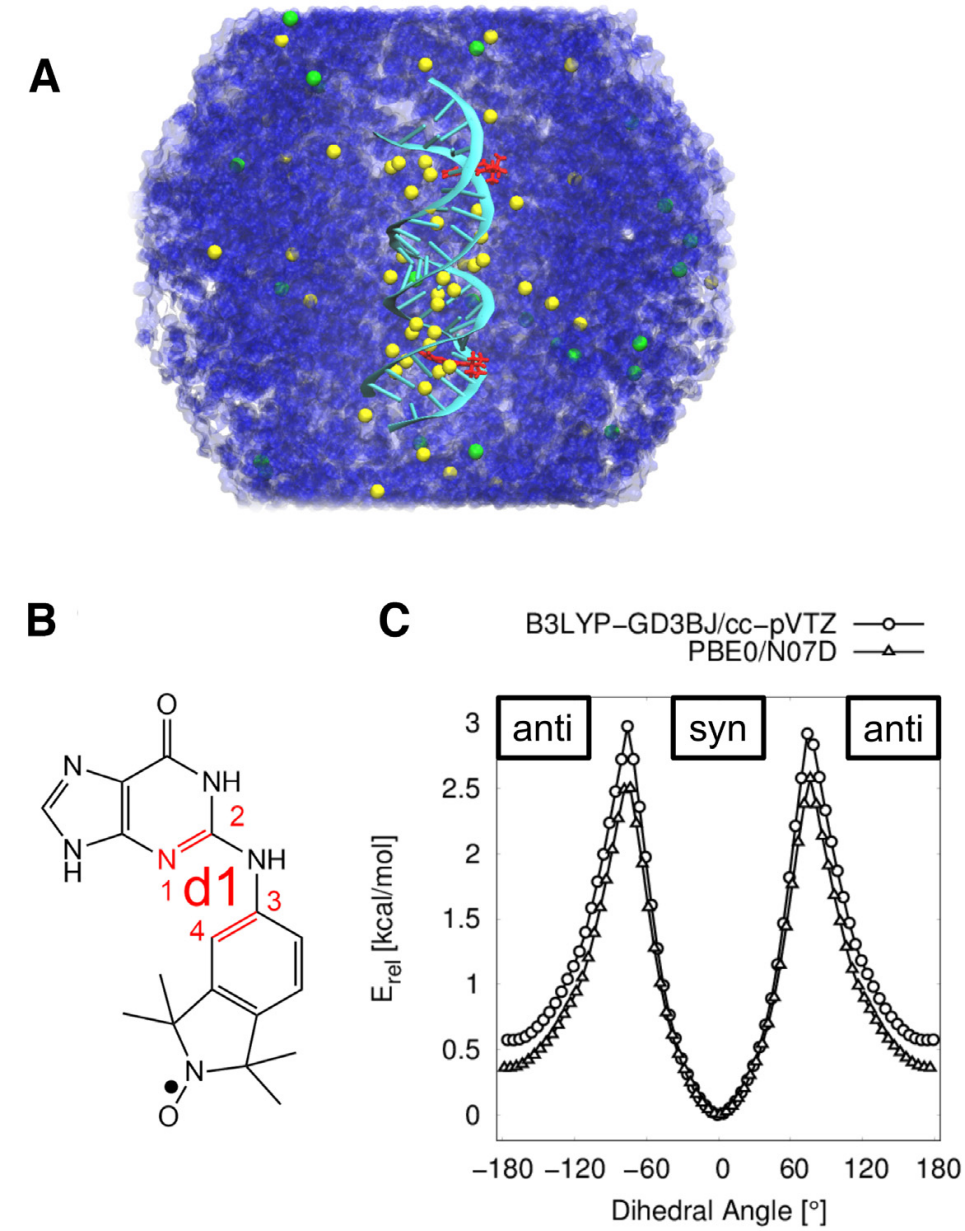
RNA MD simulations and $\bf\acute{G}$ DFT calculations. (**A**) Simulation system with two $\bf\acute{G}$ molecules (red) incorporated in the RNA helix (lime), solvated in a periodic truncated octahedral box of water (blue) with Na^+^ (yellow) and Cl^−^ ions (green). (**B**) Structure of the $\bf\acute{G}$ molecule corresponding to a dihedral angle **d1** of 0° (syn). (**C**) DFT relaxed surface scans of the dihedral angle **d1** in the $\bf\acute{G}$ molecule at B3LYP-GD3BJ/cc-pVTZ and PBE0/N07D levels of theory. Energies are relative to the minimum energy structure of each scan.

### Computational details

Relaxed surface scans of the dihedral angle **d1** in the $\bf\acute{G}$ molecule (Figure 1B) were performed using the Gaussian09 program package (36) at a PBE0/N07D (37,38) level of theory, which was used in previous nitroxide studies (1). An additional relaxed surface scan was performed with a dispersion correction (GD3BJ (39)), an increased basis set of triple-ζ quality (cc-pVTZ (40)) and the more commonly used B3LYP (41) functional. The dihedral angle between the aromatic purine scaffold and the isoindoline nitroxide plane was scanned with a step size of 5 degrees in forward and backward direction. The final energy surface reports the lowest energy for a given point in forward and backward directions.

MD simulations were performed with the Amber16 program package (42). Unlabeled nucleic acid helices were constructed with the Nucleic Acid Builder (NAB (43)). Double-stranded RNA with a palindromic sequence (5′-CGAGGAUCGCGCGCGAUCCUCG-3′) was built in the characteristic A-helical form. The underlined G represents the position of the abasic site. The dsDNA was constructed in B-helical form with an additional T base overhang at the 5′ end of each strand (i.e. 5′-TGTCAGTCGCGCGCGCGCATC-3′ and 5′-TGATGCGCGCGCGCGACTGAC-3′). Partial charges of the abasic site were newly determined, based on a restrained electrostatic potential fit (RESP (44)) as implemented in the R.E.D. Tools Version III.52 (45) (RESP-A1: HF/6-31G* Connolly surface algorithm, two-stage RESP fit qwt = 0.0005/0.001, charge value accuracy ± 10^−4^ e). The abasic site was saturated with a methoxy group at the phosphate group at the 5′ end. An additional monomethyl phosphate group was attached at the 3′ oxygen, resulting in a net charge of –2 e. The partial charges of the abasic moiety atoms were determined and the additional attached substituents removed, afterwards. To obtain a net charge of –e at the abasic site, the remaining charges were distributed over all atoms in this residue. The new partial charges were assigned to the abasic site (Supplementary Tables S1, S2). Atom types, bond-, angle- and dihedral parameters for the abasic sites in DNA were taken from the ParmBSC1 force field (46) and for the abasic sites in RNA from the ParmBSC0 force field including the χ_*OL*3_ correction (47–49). Native residues in DNA were described with the ParmBSC1 force field and native residues in RNA were described with the ParmBSC0+χ_*OL*3_ force field. The GAFF (50) parameters were assigned to the $\bf\acute{G}$ molecule via acpype (51). Partial charges of $\bf\acute{G}$ were newly derived (Supplementary Table S3), based on the RESP fitting procedure (52).

The MD systems were prepared with the tleap module as part of the AmberTools14 program package (53). A layer of at least 15 Å TIP3P water (54) molecules separated the solute from the edges of the periodic, truncated octahedral box. The systems were neutralized and additional NaCl (55) was added to mimic a salt concentration of 100 mM. The system contained ∼60 000 atoms in the RNA systems and ∼68 000 atoms in the DNA systems.

After energy minimization and equilibration (Supplementary Information), production runs were performed using the pmemd engine in Amber16. Trajectories of 1 μs (unlabeled) and 2 μs (labeled) were simulated for RNA and DNA, respectively, in 10 ns segments, where coordinates, velocities and box information were taken from the previous run. Unobserved conformational states of the $\bf\acute{G}$ molecules were individually simulated for 500 ns, i.e. anti–syn and syn–syn for RNA and syn–anti for DNA. The coordinates were wrapped into a primary box. Temperature was kept at 300 K using Langevin dynamics (γ = 1 ps^−1^). To prevent ‘synchronization’ artifacts (56), caused by the thermostat, a random seed was set at every restart. Covalent bond lengths of hydrogen atoms were maintained with the SHAKE (57) algorithm. The pressure was kept at 1 atm with isotropic position scaling and a relaxation time of 2 ps for the Berendsen barostat (58).

Due to the limited number of atom types in the general amber force field (GAFF), the $\bf\acute{G}$ nitroxide atoms (N–O) are not optimally described, i.e. the oxygen atom was always out-of the isoindoline plane during the MD simulations. In the PELDOR signal calculations, the oxygen was therefore virtually positioned in the isoindoline plane. PELDOR time traces were calculated according to Marko *et al.* (59). The following spin parameters for $\bf\acute{G}$ were used: *g* = (2.0088, 2.0065, 2.0027) and nitrogen hyperfine coupling of *A*[MHz] = (15, 15, 98). These values were validated with a continuous wave measurement of the $\bf\acute{G}$ sample (24). The modulation depth variations between the set of X-band time traces measured at different offsets were not fitted. However, for the G-band data, due to uncertainties of the resonator performance and non-perfect calculations of the excitation profiles, the modulation depth was adjusted for each time trace. To simulate the PELDOR signal, 20 000 structures (every 0.1 ns) were taken from the respective 2 μs MD trajectories and 2000 equally spaced structures for every $\bf\acute{G}$ conformational state (syn–syn, anti–syn, syn–anti and anti–anti).

Helical and base pair parameters were determined with do_x3DNA (60,61) for GROMACS, after converting the trajectories from .ntx format to .pdb format with VMD (62) version 1.9.2.

## RESULTS AND DISCUSSION

### Characterization of $\bf\acute{G}$

The spin label $\bf\acute{G}$ (24) is comprised of an isoindoline derivative and an aminoxyl radical, and an aromatic purine scaffold, connected by a rotatable amine bridge (Figure 1B). DFT relaxed surface scans of the dihedral angle **d1** (bonds 1–2 and 3–4 connected by pseudo-bond 2–3 in Figure 1B) at B3LYP-GD3BJ/cc-pVTZ and PBE0/N07D levels of theory (Figure 1C) reveal a global minimum at 0° (syn) and a local minimum at 180° (anti), indicating two favored conformations of the $\bf\acute{G}$ molecule in the gas phase without the nucleic acid. A detailed discussion of the conformational landscape of $\bf\acute{G}$ can be found in the Supporting Informations (Supplementary Figure S3).

### MD simulations of $\bf\acute{G}$-labeled DNA

**Figure 2.**
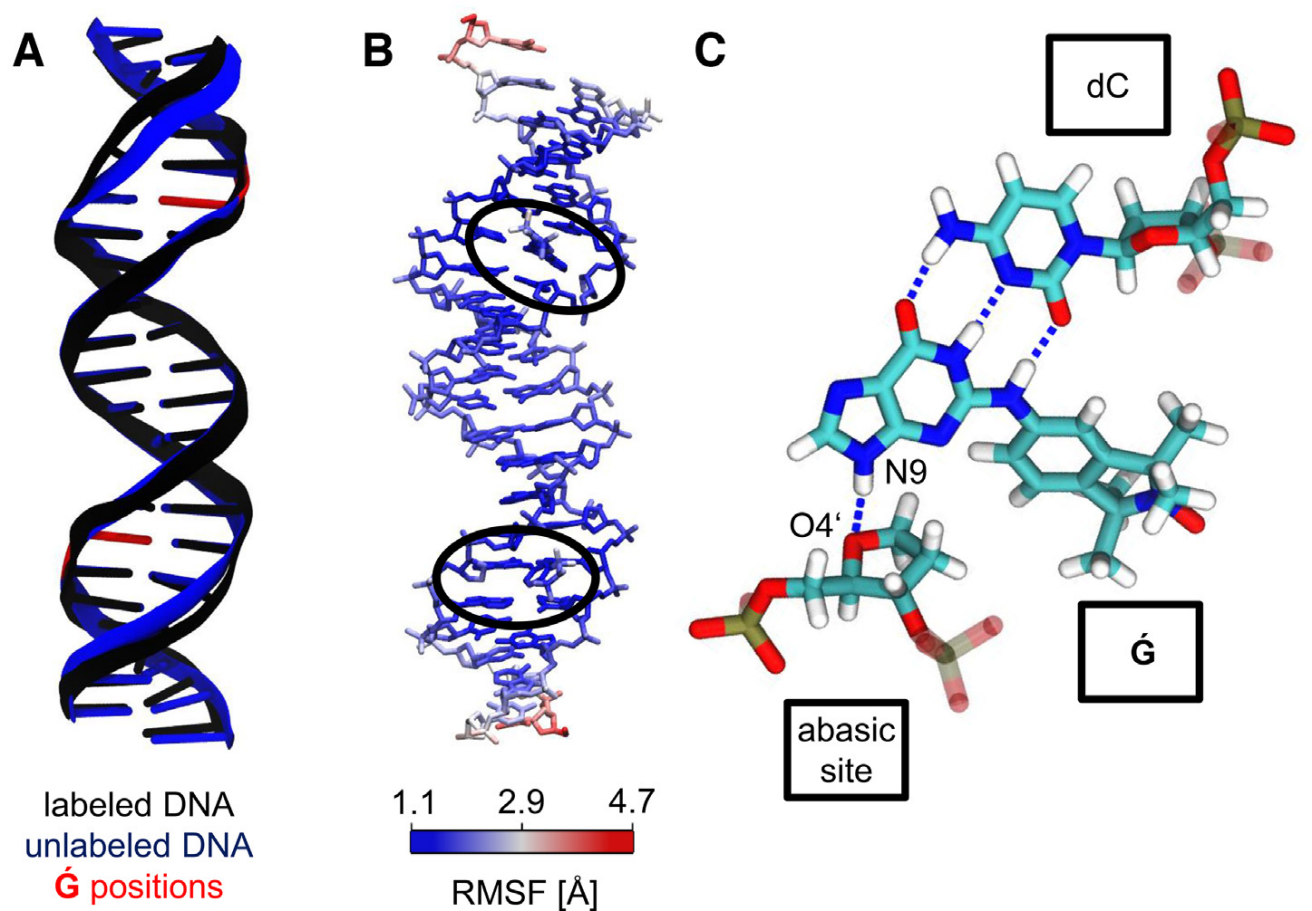
Structural influence of $\bf\acute{G}$ labeling on dsDNA. (**A**) Average structures of the labeled and unlabeled dsDNA simulations. The position of the $\bf\acute{G}$ molecules and abasic sites are depicted in red at the unlabeled structure. (**B**) Atom position root mean square fluctuations of $\bf\acute{G}$-labeled dsDNA, color coded on a representative MD structure. The averaged structure of the labeled dsDNA simulation was taken as the reference state. $\bf\acute{G}$ regions are highlighted with black ellipses. (**C**) $\bf\acute{G}$ interactions in a dsDNA. Stable hydrogen bonds between $\bf\acute{G}$ and the complementary dC are shown as dashed lines. An additional hydrogen bond forms between $\bf\acute{G}$ (N9) and the abasic site (O4′).

We then proceeded to study the conformations of $\bf\acute{G}$ in a dsDNA helix. In MD simulations of 2 μs, the two $\bf\acute{G}$ labels remained in the abasic sites of the dsDNA. During these 2 μs of MD simulation the dsDNA kept its helical structure, as indicated by the averaged structure (Figure 2A). The heavy-atom position root-mean-square deviation (RMSD) of the $\bf\acute{G}$-labeled dsDNA from an ideal B-helical structure was only 1 Å larger than for an unlabeled dsDNA simulation (unlabeled: 2.9 ± 0.6 Å, labeled: 3.9 ± 0.7 Å). The atom position root-mean-square fluctuation (RMSF) of the labeled dsDNA trajectory, aligned onto the averaged structure, revealed base flexibility at the 5′-ends and 3′-ends of each strand, as expected (Figure 2B and Supplementary Figure S4). Slightly elevated flexibility was also observed at the abasic sites occupied by the $\bf\acute{G}$ molecules. The purine end of each $\bf\acute{G}$ molecule remained at the position of the native guanine base and formed hydrogen bonds with the corresponding cytidine in the complementary strand, mimicking native bases (Figure 2C). An additional hydrogen bond was observed between the nitrogen (N9) of the $\bf\acute{G}$ molecules and the oxygen (O4′) of the abasic site. The isoindoline aminoxyl radical end of the $\bf\acute{G}$ molecule was positioned inside the minor groove of the helix.

The abasic sites and $\bf\acute{G}$ molecules slightly perturbed the helical dsDNA structure. Individual averages over backbone torsion angles were calculated for an unlabeled dsDNA and the complementary $\bf\acute{G}$-labeled dsDNA. The difference between the torsion angles reveal changes in the ζ torsion angle of up to 42° close to the spin label position and –25° directly at the spin label position (Supplementary Figure S5). The δ torsion angle of the corresponding cytidine in the complementary strand was also perturbed with deviations up to 34°. Visual inspection of the MD trajectory revealed that the abasic site sometimes rotates slightly out of a native-like helix structure. However, the overall helical structure stayed intact during these short events (Figure 2A).

**Figure 3.**
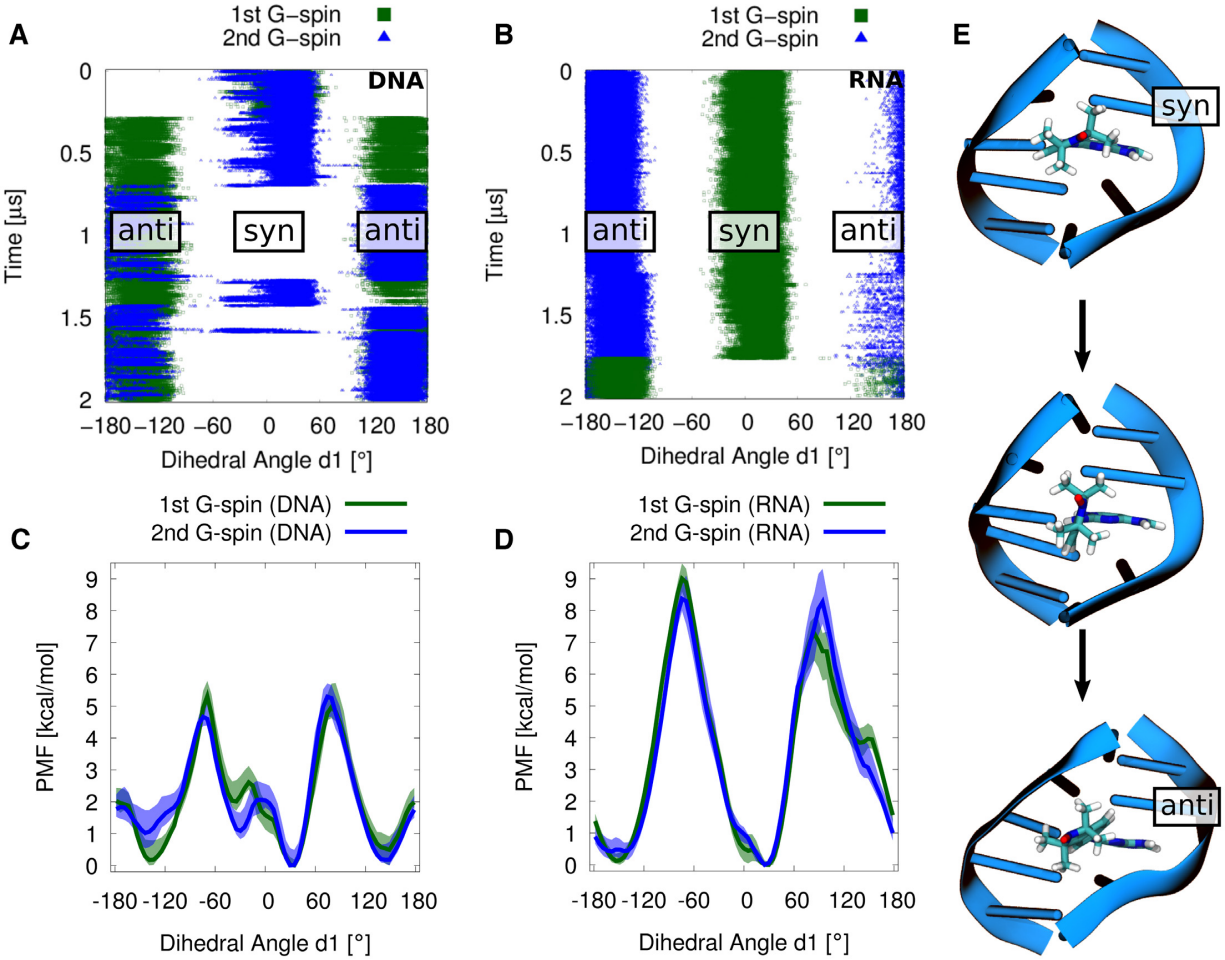
(**A**, **B**) Dihedral angle **d1** between the aromatic purine scaffold and the isoindoline nitroxide plane of the two $\bf\acute{G}$s in dsDNA and dsRNA during the MD simulations. (**C**, **D**) Potential of mean force profiles for rotations of $\bf\acute{G}$ inside the minor grooves of dsDNA and dsRNA. Note that the dsDNA sequence is not palindromic, unlike the dsRNA sequence, explaining the small difference between the two profiles in C. Shaded areas represent the minimum and maximum values of blocking analysis, using 10 blocks. (**E**) Rotation from syn to anti conformation of dihedral angle **d1**. $\bf\acute{G}$ is shown as sticks, and surrounding DNA bases are shown as cartoon.

With the MD simulations, we resolved the possible spin label orientations and their relative frequency. In the MD simulation, several rotations around the dihedral angle **d1** in both $\bf\acute{G}$ molecules were observed (Figure 3A), capturing three out of four possible states, i.e. (i) both $\bf\acute{G}$ molecules in syn (syn–syn), (ii) one molecule in anti (anti–syn) and (iii) both spin labels in anti configuration conformation (anti–anti). A syn to anti rotation inside the minor groove placed the isoindoline plane parallel to the backbone (Figure 3E). An additional simulation of 500 ns in the missing (iv) syn–anti conformation was performed for further distance distribution analysis.

To check that the accessible conformational space was explored in the MD simulations, we performed umbrella sampling calculations of the rotations around the dihedral angle **d1** (Figure 3C). The reported uncertainties correspond to the range between the minimum and maximum values of the free energy profiles obtained by blocking the data into 10 segments that were analyzed individually (Supplementary Figure S6). The potential of mean force (PMF) profiles follow a similar pattern for both $\bf\acute{G}$ molecules (Figure 3C). For $\bf\acute{G}$ in dsDNA, we found three free energy minima: at –140°, 30° and 150°. We also observed an additional substate at –35°. Note that the force field employed for the $\bf\acute{G}$ only approximately describes the reference behavior as based on DFT calculations in vacuo at the syn and anti minima (Supplementary Figure S7). To be consistent with the DFT calculations, we will below refer to the two states at 30° and –35° as a single syn state, and to the two states at –140° and 150° as a single anti state. The syn and anti conformers are separated by barriers of ∼5 kcal·mol^−1^. The small difference between the two $\bf\acute{G}$ PMF profiles is presumably caused by a flexibility in one of the abasic sites in the non-palindromic dsDNA. Nonetheless, the PMF profiles reveal that all possible states are captured by the MD simulations.

### Comparison to PELDOR experiments of $\bf\acute{G}$-labeled DNA

**Figure 4.**
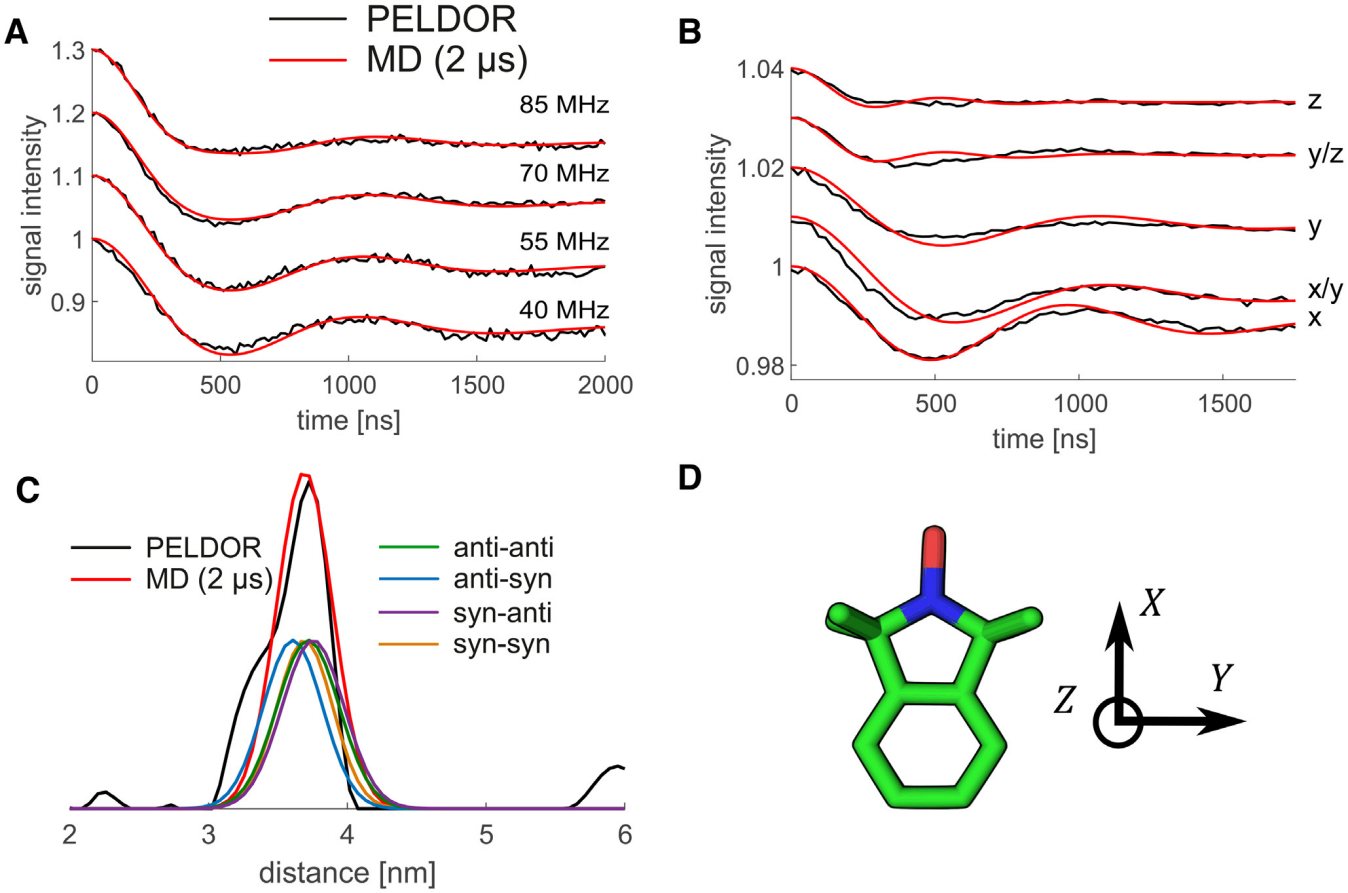
Comparison of PELDOR signals calculated directly from MD (red) to experiments (black) for DNA(1,10) without any fit. (**A**) PELDOR time traces measured at X-band with different offsets ν_*n*_ between probe and pump pulse frequency from 40 MHz (lower time trace) to 85 MHz (upper time trace). (**B**) PELDOR time traces measured at G-band at different field positions with a constant offset of 60 MHz. The field position corresponds to *B*∥*g*_*xx*_ − *B*∥*g*_*zz*_. (**C**) Distance distribution from PELDOR measurements obtained by Tikhonov regularization (black) and from MD simulation (red), including the individual conformational states of the $\bf\acute{G}$ molecule. The contribution of the different syn and anti combinations are shown as well. (**D**) Axis system of a nitroxide spin label.

Both the low-field X-band and the high-field G-band PELDOR time traces calculated for the dsDNA MD simulation structures are in excellent agreement with the measured traces (Figure 4A, B). The PELDOR time traces were calculated directly and without adjustable parameters from the coordinates of the two spin labels in the MD trajectories (1,33), and then compared with the experimental PELDOR time traces. The low-field X-band (0.3 T/9 GHz) EPR spectra are dominated by the nitrogen hyperfine anisotropy, which is an axial tensor with its large value parallel to the *z*-axis (normal to the nitroxide plane, Figure 4D). As can be seen in Figure 4A, hardly any orientation selectivity is visible in the X-band PELDOR time traces collected at different probe frequencies. An inspection of the MD simulation structures shows that this lack of orientation selectivity arises from the fact that the angles between the *z*-axis and the inter-spin vector cluster ∼40°. Such orientations do not exhibit orientation dependence at X-band frequencies (Supplementary Figure S8). Using state-of-the-art DNA force fields, MD simulations thus capture the structure and motions not only of regular B-helix DNA (1), but also of dsDNA with abasic sites occupied by large base analogs. In addition, the simulations rationalize the absence of orientation selectivity in the X-band PELDOR measurements.

By contrast, orientation selectivity is pronounced at G-band frequencies, resulting in differences in oscillation frequency, oscillation dampening, and modulation depth as a function of magnetic field strength (Figure 4B). This orientation selectivity is captured by the PELDOR time traces calculated from dsDNA MD trajectories, which are in excellent agreement with the measurements. High-field G-band (180 GHz/6.4 T) EPR spectra are dominated by the *g*-tensor anisotropy, giving us information on the in-plane orientation (with respect to the *x* and *y* axes, Figure 4D). The possible 180° rotation around dihedral angle **d1** of the spin labels in the abasic binding site do not alter the relative spin orientations, due to the 180° symmetry of all spin interactions with respect to the direction of the magnetic field. Therefore, such rotations around **d1**, as observed in the MD trajectories, retain orientation selectivity.

We extracted the distribution of the distance between the two spin labels via Tikhonov regularization of the sum over all X-band time traces (63) (Figure 4C and Supplementary Figure S9). The experimental distribution peaks at 37.2 Å with a shoulder at 33.9 Å. The shoulder at 33.9 Å might easily arise from residual orientation effects, as the distance distribution is derived from the time trace summing up all four offsets. Previously we showed that a sum of seven time traces with offsets from 30 to 90 MHz represents very well a statistical powder sample, leading to a reliable distance distribution (63). Therefore, the shoulder should not be interpreted. The distance distribution obtained from the 2 μs long MD trajectories peaks at 36.8 Å and coincides almost exactly with the experimental distribution (Figure 4C). The PELDOR time traces of the individual conformational states of the two $\bf\acute{G}$ molecules from MD simulations were calculated (Supplementary Figure S10) and the corresponding distance distributions extracted (Figure 4C). The anti-syn conformation has the shortest distance of 36.1 Å. Anti-anti and syn-syn conformations are almost identical at 37.3 and 36.7 Å, while syn–anti results in somewhat longer distances of 37.5 Å. All these individual distances are very similar, suggesting that the experimentally observed distance distribution is determined by a combination of dsDNA helix movements, $\bf\acute{G}$ movement in the abasic site, and to a lesser degree by the distribution of $\bf\acute{G}$ conformational states. A comparison between PELDOR experiments using covalently attached **Ç** (1) and non-covalently attached $\bf\acute{G}$ spin labels for the same dsDNA sequence shows similar broadening of the inter-spin distance distributions (FWHM: **Ç** 0.633 nm, $\bf\acute{G}$: 0.675 nm) as determined by Tikhonov regularization from the experimental data (Supplementary Figure S11).

### MD simulations of $\bf\acute{G}$-labeled RNA

**Figure 5.**
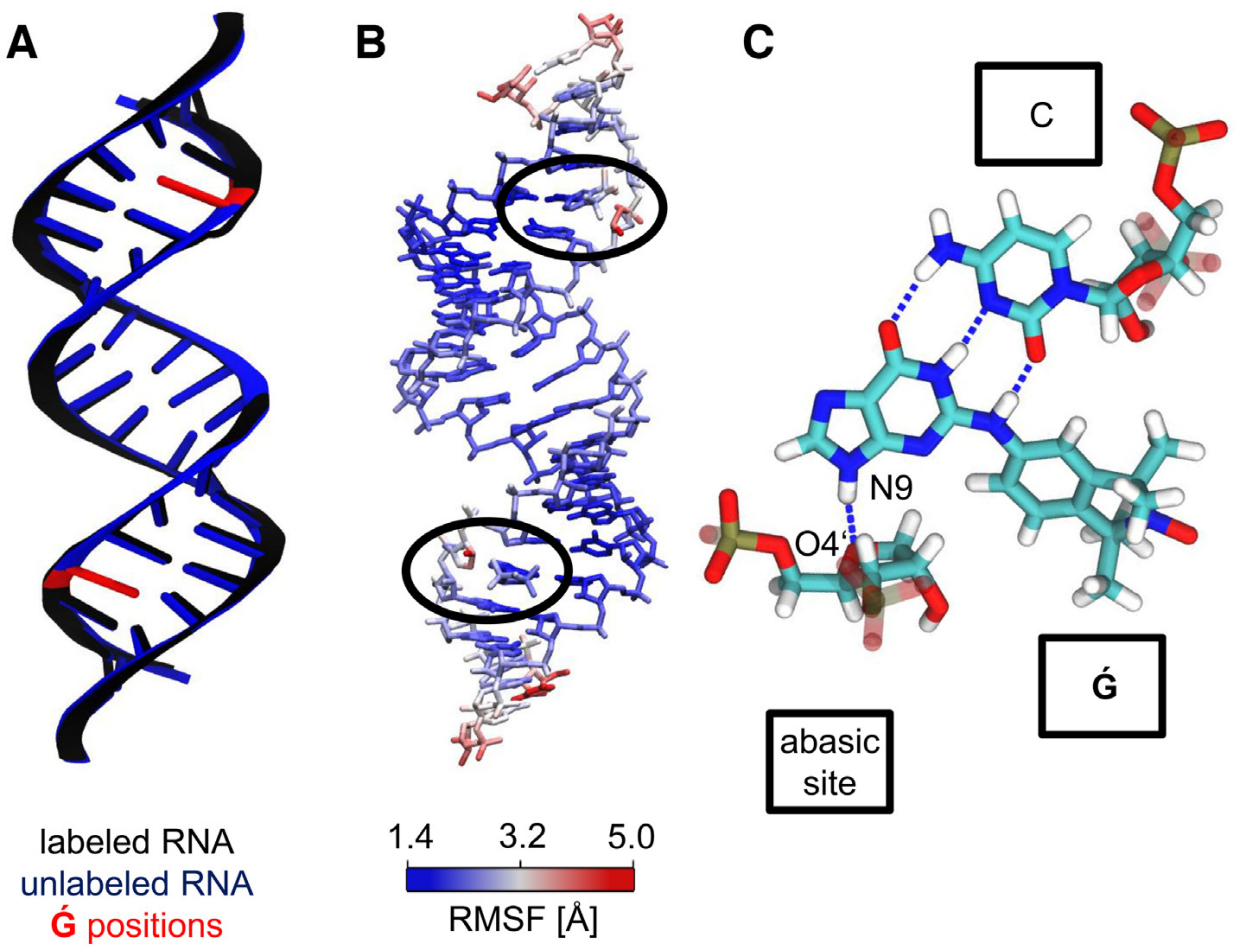
Influence of $\bf\acute{G}$ labeling on dsRNA structure. (**A**) Average structures of the labeled and unlabeled dsRNA simulations. The position of the $\bf\acute{G}$ molecules and abasic sites are depicted in red at the unlabeled structure. (**B**) Atom position root mean square fluctuations of $\bf\acute{G}$-labeled dsRNA, color coded on a representative MD structure. The averaged structure of the labeled dsRNA simulation was taken as the reference state. $\bf\acute{G}$ regions are highlighted with black ellipses. (**C**) $\bf\acute{G}$ interactions in a dsRNA. Stable hydrogen bonds between $\bf\acute{G}$ and the complementary cytidine are shown as dashed lines. An additional hydrogen bond formed between $\bf\acute{G}$ (N9) and the abasic site (O4′).

The MD simulation of two $\bf\acute{G}$ molecules non-covalently attached to a dsRNA helix revealed an intact helical structure (Figure 1A). Structurally, the $\bf\acute{G}$ molecule showed similar behavior in dsRNA as in dsDNA. The purine ends of the $\bf\acute{G}$ molecules were located inside the A-helix, forming hydrogen bonds to their complementary cytidines on the opposite strand, thereby mimicking native guanine bases (Figure 5C). An additional hydrogen bond formed between the purine nitrogen (N9) and the oxygen (O4′) of the abasic site sugar, which appeared to be less stable than in dsDNA (Supplementary Figure S12). The isoindoline aminoxyl radical end pointed into the minor groove, as already proposed previously (24).

The abasic sites occupied by $\bf\acute{G}$ molecules did not significantly affect the overall dsRNA structure. The backbone torsion angles of the dsRNA were calculated for unlabeled dsRNA and for a dsRNA with two abasic sites occupied by $\bf\acute{G}$. The differences in the torsion angles of unlabeled and labeled dsRNA revealed a small perturbation directly at the abasic sites and the corresponding cytidine in the complementary strand (Supplementary Figure S5). The overall structure stayed intact and the perturbation was negligibly small with an overall heavy atom position RMSD to an ideal A-helix of 3.4 ± 0.8 Å compared to an unlabeled dsRNA simulation with an RMSD of 2.9 ± 0.7 Å. The helical parameters of the inner, canonical base pairs of the dsRNA MD simulations of the labeled and unlabeled dsRNA agree also within statistical uncertainty (Supplementary Table S4) and are in line with helical parameters for a GC rich sequence reported by Šponer *et al.* at slightly different solution conditions (29). The averaged structure reflects these similarities and is almost indistinguishable to the averaged structure of an unlabeled dsRNA simulation (Figure 5A). The atom position RMSF of the labeled dsRNA trajectory aligned onto their averaged structure reveals flexibility at the blunt ends (Figure 5B). Both abasic sites also had higher fluctuations, due to the short rotations of the sugar moieties out of the helical structure. The $\bf\acute{G}$ molecules remained rigid inside the helix.

A single rotation of the dihedral angle **d1** of a $\bf\acute{G}$ molecule was observed in dsRNA within 2 μs of MD simulation, transitioning from (i) syn–anti to (ii) anti–anti states (Figure 3B). The unresolved $\bf\acute{G}$ conformational states, i.e. (iii) syn–syn and (iv) anti–syn, were sampled in additional MD simulations of 500 ns duration, each started in these two states. To rule out missing conformations, an umbrella sampling of **d1** was performed (Figure 3D). The resulting PMF profiles are similar for the two sites, revealing two minima at –150° and 30°. The similarity of the profiles provides some confidence in the accuracy of the umbrella sampling calculations, as the RNA sequence we studied is palindromic (unlike the DNA sequence). Barriers of about ∼8 kcal·mol^−1^ separate the dominant minima. The higher barriers, compared to rotations in dsDNA (5 kcal·mol^−1^), are in line with the observed single rotation of the $\bf\acute{G}$ molecule in the dsRNA MD simulation and the more frequent rotations of $\bf\acute{G}$ in dsDNA within the same simulation time of 2 μs.

### Comparison to PELDOR experiments of $\bf\acute{G}$-labeled RNA

**Figure 6.**
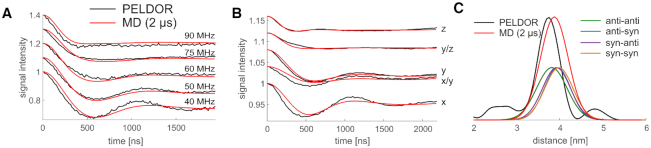
Comparison of PELDOR signals calculated from MD (red) to experiments (black) for RNA(1,10). (**A**) PELDOR time traces measured at X-band with different offsets ν_*n*_ between probe and pump pulse frequency from 40 MHz (lower time trace) to 90 MHz (upper time trace). (**B**) PELDOR time traces measured at G-band at different field positions with a constant offset of 60 MHz. The field position correspond to *B*∥*g*_*xx*_ − *B*∥*g*_*zz*_ (**C**) Distance distribution from PELDOR measurements obtained by Tikhonov regularization (black), and from MD simulation (red), including the individual conformational states of the $\bf\acute{G}$ molecules.

Average PELDOR time traces were calculated from the dsRNA MD simulations separately for each individual conformational state (Supplementary Figure S13) and for the 2 μs MD simulation (Figure 6A, B). Additionally, we averaged the PELDOR time traces of the four conformational states with equal weights (Supplementary Figure S14), as indicated by the calculated PMF profile (Figure 3D). We noticed that the differences between the calculated PELDOR time traces of the individual states, the PELDOR time traces averaged over all states, and the PELDOR time traces of the 2 μs MD simulation are very small and that the main characteristics are preserved. Therefore, we focused on the PELDOR time traces of the 2 μs MD simulation to be consistent with the dsDNA comparison.

Comparisons of measured and calculated PELDOR time traces and of the inter-spin distances for labeled dsRNA show very good agreement overall (Figure 6). Excellent agreement was achieved for the low-field X-band data (Figure 6A). The experimental PELDOR time traces (X-band) revealed differences in the damping, which give strong evidence for a highly preserved out-of-plane orientation of the $\bf\acute{G}$ in dsRNA (Figure 6A). In the high-field G-band data, the PELDOR experiments showed pronounced orientation selectivity (Figure 6B), with good agreement between the in-plane orientations in the measured and calculated PELDOR time traces. The extracted distance population of the experimental PELDOR data peaks at 37.5 Å, whereas the directly extracted distances from the MD simulation tend to somewhat longer distances, with a main population at 38.7 Å (Figure 6C). We note that the differences in mean spin-spin distance of 1–2 Å are similar to what we obtained previously by comparing MD simulations of dsDNA to PELDOR experiments with the covalently attached and rigid **Ç** (1). Somewhat larger differences were reported by Halbmair *et al.* (32) in a comparison between MD simulations of dsRNA and experiments with a more flexible spin label. Indeed, earlier MD simulations of dsRNA with the ParmBSC0 + χ_*OL*3_ force field and TIP3P water model (29) indicated a possible bias towards somewhat elongated dsRNA A-helices for GC rich sequences. However, more experiments, with additional spin-label pairs would be required to test whether A-helices are systematically elongated in the simulations.

The individual distance distributions of the four conformational states of the two $\bf\acute{G}$ molecules were calculated and largely overlap. An anti-anti conformation has the shortest spin-spin distance with 37.8 Å. The syn–syn conformation results in the longest distances with 39.8 Å, while syn–anti and anti–syn are indistinguishable in between (39.5 Å). The overlap in the distance distribution between the different states suggests that the overall distance distribution is mostly affected by the global helix movement, rather than by the conformational state of $\bf\acute{G}$. Indeed, the distance distributions of the syn–syn, syn–anti, anti–syn and anti–anti states have nearly the same width as the overall distribution (Figure 6C). The comparison to PELDOR experiments, which report on long-range distances and angles, shows that the structure and motions of RNA A-helices are described well by the ParmBSC0 + χ_*OL*3_ force field.

## CONCLUSIONS

The comparison between MD simulations and PELDOR experiments reveals that the $\bf\acute{G}$ label is well suited for distance measurements using PELDOR in both dsDNA and dsRNA. The simulated $\bf\acute{G}$ molecule distance distributions are on par with the PELDOR experiment in dsDNA and in excellent agreement for dsRNA. In both labeled nucleic acid helices, the helical structure remained intact. Local perturbations were introduced into the system near the abasic sites and the non-covalently bound $\bf\acute{G}$ molecules, which were very small in dsDNA and almost negligible in dsRNA. Watson–Crick hydrogen bonding kept the $\bf\acute{G}$ molecules at their corresponding purine positions. In bound $\bf\acute{G}$, rotations around the dihedral angle **d1** were more frequently observed in dsDNA than in dsRNA. Interestingly, the non-covalently attached $\bf\acute{G}$ molecule did not significantly affect the width of the distance distribution in comparison to the completely rigid and covalently attached spin label **Ç** (1), which requires a much higher synthetic effort for incorporation into nucleic acids.

The direct comparison to PELDOR measurements provides highly accurate information to evaluate current state-of-the-art force fields for nucleic acids. Both force fields, ParmBSC1 for DNA and ParmBSC0 + χ_*OL*3_ for RNA, are able to describe double-stranded, helical nucleic acids well. For dsDNA there is almost no difference discernible between the experimental PELDOR data and PELDOR time traces computed from the MD simulation. The excellent agreement for ParmBSC1 confirms our previous conclusion that state-of-the-art force fields (including OL15 (64–66)) describe the structure of dsDNA well (1). By contrast, the discrepancies between experiment and simulation were somewhat larger for dsRNA, though still small. The well-established ParmBSC0 + χ_*OL*3_ force field for RNA seems to describe the structure of dsRNA well. The MD inter-spin distance distribution is slightly shifted by 1.2 Å toward longer distances, presumably caused by a slight elongation of the helix, which may be associated with the choice of the water model (29). Still, the calculated PELDOR time traces from the MD simulations match well with the PELDOR data, but further improvement is possible. As new RNA force fields are developed, we envisage that PELDOR data will provide a valuable reference for validation by providing highly accurate long-range distance information.

## Supplementary Material

gkz1096_Supplemental_File

## References

[1] Stelzl L.S., Erlenbach N., Heinz M., Prisner T.F., Hummer G. Resolving the conformational dynamics of DNA with Ångstrom resolution by pulsed electron-electron double resonance and molecular dynamics. J. Am. Chem. Soc. 2017; 139:11674–11677. 28777549 10.1021/jacs.7b05363

[2] Milov A.D., Ponomarev A.B., Tsvetkov Y.D. Electron-electron double resonance in electron spin echo: model biradical systems and the sensitzed photolysis of decalin. Lett. Chem. Phys. 1984; 110:67–72.

[3] Peselis A., Gao A., Serganov A. Preparation and crystallization of riboswitches. Methods Mol. Biol. 2016; 1320:21–36. 26227035 10.1007/978-1-4939-2763-0_3

[4] Nozinovic S., Fürtig B., Jonker H.R.A., Richter C., Schwalbe H. High-resolution NMR structure of an RNA model system: the 14-mer cUUCGg tetraloop hairpin RNA. Nucleic Acids Res. 2010; 38:683–694. 19906714 10.1093/nar/gkp956 PMC2811024

[5] Fürtig B., Richter C., Wöhnert J., Schwalbe H. NMR spectroscopy of RNA. ChemBioChem. 2003; 4:936–962. 14523911 10.1002/cbic.200300700

[6] Hennig J., Sattler M. The dynamic duo: combining NMR and small angle scattering in structural biology. Protein Sci. 2014; 23:669–682. 24687405 10.1002/pro.2467 PMC4093944

[7] Bai X.-c., Martin T.G., Scheres S.H.W., Dietz H. Cryo-EM structure of a 3D DNA-origami object. Proc. Natl. Acad. Sci. U.S.A. 2012; 109:20012–20017. 23169645 10.1073/pnas.1215713109 PMC3523823

[8] Topf M., Lasker K., Webb B., Wolfson H., Chiu W., Sali A. Protein structure fitting and refinement guided by Cryo-EM density. Structure. 2008; 16:295–307. 18275820 10.1016/j.str.2007.11.016 PMC2409374

[9] Diskowski M., Mehdipour A.R., Wunnicke D., Mills D.J., Mikusevic V., Bärland N., Hoffmann J., Morgner N., Steinhoff H.-J., Hummer G. et al. Helical jackknives control the gates of the double-pore K^+^ uptake system KtrAB. eLife. 2017; 6:e24303. 28504641 10.7554/eLife.24303 PMC5449183

[10] Ding Y., Zhang X., Tham K.W., Qin P.Z. Experimental mapping of DNA duplex shape enabled by global lineshape analyses of a nucleotide-independent nitroxide probe. Nucleic Acids Res. 2014; 42:e140. 25092920 10.1093/nar/gku695 PMC4191381

[11] Krumkacheva O.A., Shevelev G.Y., Lomzov A.A., Dyrkheeva N.S., Kuzhelev A.A., Koval V.V., Tormyshev V.M., Polienko Y.F., Fedin M.V., Pyshnyi D.V. et al. DNA complexes with human apurinic/apyrimidinic endonuclease 1: structural insights revealed by pulsed dipolar EPR with orthogonal spin labeling. Nucleic Acids Res. 2019; 47:7767–7780. 31329919 10.1093/nar/gkz620 PMC6735896

[12] Endeward B., Butterwick J.A., MacKinnon R., Prisner T.F. Pulsed electron-electron double-resonance determination of spin-label distances and orientations on the tetrameric potassium ion channel KcsA. J. Am. Chem. Soc. 2009; 131:15246–15250. 19919160 10.1021/ja904808n PMC2779547

[13] Reichel K., Stelzl L.S., Köfinger J., Hummer G. Precision DEER distances from spin-label ensemble refinement. J. Phys. Chem. Lett. 2018; 9:5748–5752. 30212206 10.1021/acs.jpclett.8b02439

[14] Barhate N., Cekan P., Massey A.P., Sigurdsson S.T. A nucleoside that contains a rigid nitroxide spin label: a fluorophore in disguise. Angew. Chem. 2007; 119:2709–2712. 10.1002/anie.200603993 17309085

[15] Sicoli G., Wachowius F., Bennati M., Höbartner C. Probing secondary structures of spin-labeled RNA by pulsed EPR spectroscopy. Angew. Chem. Int. Ed. 2010; 49:6443–6447. 10.1002/anie.201000713 20665607

[16] Shevelev G.Y., Krumkacheva O.A., Lomzov A.A., Kuzhelev A.A., Trukhin D.V., Rogozhnikova O.Y., Tormyshev V.M., Pyshnyi D.V., Fedin M.V., Bagryanskaya E.G. Triarylmethyl labels: toward improving the accuracy of EPR nanoscale distance measurements in DNAs. J. Phys. Chem. B. 2015; 119:13641–13648. 26011022 10.1021/acs.jpcb.5b03026 PMC4824051

[17] Haugland M.M., Anderson E.A., Lovett J.E. Tuning the properties of nitroxide spin labels for use in electron paramagnetic resonance spectroscopy through chemical modification of the nitroxide framework. Electron Paramagn. Reson. 2017; 25:1–34.

[18] Kerzhner M., Abdullin D., Wicek J., Matsuoka H., Hagelueken G., Schiemann O., Famulok M. Post-synthetic spin-labeling of RNA through click chemistry for PELDOR measurements. Chem. - A Eur. J. 2016; 22:12113–12121. 10.1002/chem.201601897 27412453

[19] Gophane D.B., Endeward B., Prisner T.F., Sigurdsson S.T. A semi-rigid isoindoline-derived nitroxide spin label for RNA. Org. Biomol. Chem. 2018; 16:816–824. 29326999 10.1039/c7ob02870a

[20] Hong S., Piette L.H. Electron spin resonance spin-label studies of intercalation of ethidium bromide and aromatic amine carcinogens in DNA. Cancer Res. 1976; 36:1159–1171. 1253173

[21] Belmont P., Chapelle C., Demeunynck M., Michon J., Michon P., Lhomme J. Introduction of a nitroxide group on position 2 of 9-phenoxyacridine: Easy access to spin labelled DNA-binding conjugates. Bioorg. Med. Chem. Lett. 1998; 8:669–674. 9871580 10.1016/s0960-894x(98)00089-4

[22] Shelke S.A., Sigurdsson S.T. Site-directed spin labelling of nucleic acids. Eur. J. Org. Chem. 2012; 2012:2291–2301.

[23] Shelke S.A., Sigurdsson S. Th. Structural changes of an abasic site in duplex DNA affect noncovalent binding of the spin label ç. Nucleic Acids Res. 2011; 40:3732–3740. 22210856 10.1093/nar/gkr1210 PMC3333849

[24] Kamble N.R., Gränz M., Prisner T.F., Sigurdsson S.T. Noncovalent and site-directed spin labeling of duplex RNA. Chem. Commun. 2016; 52:14442–14445. 10.1039/c6cc08387k 27901530

[25] Dans P.D., Ivani I., Hospital A., Portella G., González C., Orozco M. How accurate are accurate force-fields for B-DNA?. Nucleic Acids Res. 2017; 45:4217–4230. 28088759 10.1093/nar/gkw1355 PMC5397185

[26] Seyfried P., Heinz M., Pintér G., Klötzner D.-P., Becker Y., Bolte M., Jonker H.R.A., Stelzl L.S., Hummer G., Schwalbe H. et al. Optimal destabilization of DNA double strands by single-nucleobase caging. Chem. Eur. J. 2018; 24:17568–17576. 30199112 10.1002/chem.201804040

[27] Grotz K.K., Nueesch M.F., Holmstrom E.D., Heinz M., Stelzl L.S., Schuler B., Hummer G. Dispersion correction alleviates dye stacking of single-stranded DNA and RNA in simulations of single-molecule fluorescence experiments. J. Phys. Chem. B. 2018; 122:11626–11639. 30285443 10.1021/acs.jpcb.8b07537

[28] Mlýnský V., Banáš P., Hollas D., Réblová K., Walter N.G., Šponer J., Otyepka M. Extensive molecular dynamics simulations showing that canonical G8 and protonated A38H+ forms are most consistent with crystal structures of hairpin ribozyme. J. Phys. Chem. B. 2010; 114:6642–6652. 20420375 10.1021/jp1001258 PMC2872159

[29] Beššeová I., Banáš P., Kührová P., Košinová P., Otyepka M., Šponer J. Simulations of a-RNA duplexes. The effect of sequence, solute force field, water model, and salt concentration. J. Phys. Chem. B. 2012; 116:9899–9916. 22809319 10.1021/jp3014817

[30] Liebl K., Drsata T., Lankas F., Lipfert J., Zacharias M. Explaining the striking difference in twist-stretch coupling between DNA and RNA: a comparative molecular dynamics analysis. Nucleic Acids Res. 2015; 43:10143–10156. 26464435 10.1093/nar/gkv1028 PMC4666353

[31] Šponer J., Bussi G., Krepl M., Banáš P., Bottaro S., Cunha R.A., Gil-Ley A., Pinamonti G., Poblete S., Jurečka P. et al. RNA structural dynamics as captured by molecular simulations: a comprehensive overview. Chem. Rev. 2018; 118:4177–4338. 29297679 10.1021/acs.chemrev.7b00427 PMC5920944

[32] Halbmair K., Seikowski J., Tkach I., Höbartner C., Sezer D., Bennati M. High-resolution measurement of long-range distances in RNA: pulse EPR spectroscopy with TEMPO-labeled nucleotides. Chem. Sci. 2016; 7:3172–3180. 29997809 10.1039/c5sc04631a PMC6005265

[33] Marko A., Margraf D., Cekan P., Sigurdsson S.T., Schiemann O., Prisner T.F. Analytical method to determine the orientation of rigid spin labels in DNA. Phys. Rev. E. 2010; 81:021911. 10.1103/PhysRevE.81.021911 20365599

[34] Denysenkov V.P., Prisner T.F., Stubbe J., Bennati M. High-frequency 180 GHz PELDOR. Appl. Magn. Reson. 2005; 29:375–384.

[35] Jeschke G., Chechik V., Ionita P., Godt A., Zimmermann H., Banham J., Timmel C.R., Hilger D., Jung H. DeerAnalysis2006-a comprehensive software package for analyzing pulsed ELDOR data. Appl. Magn. Reson. 2006; 30:473–498.

[36] Frisch M.J., Trucks G.W., Schlegel H.B., Scuseria G.E., Robb M.A., Cheeseman J.R., Scalmani G., Barone V., Mennucci B., Petersson G.A. et al. Gaussian 09 , Revision D.01. 2009; Wallingford, CT. Gaussian, Inc.

[37] Adamo C., Barone V. Toward reliable density functional methods without adjustable parameters: the PBE0 model. J. Chem. Phys. 1999; 110:6158–6170.

[38] Barone V., Cimino P. Validation of the B3LYP/N07D and PBE0/N07D computational models for the calculation of electronic g-tensors. J. Chem. Theory Comput. 2009; 5:192–199. 26609832 10.1021/ct800279g

[39] Grimme S., Ehrlich S., Goerigk L. Effect of the damping function in dispersion corrected density functional theory. J. Comput. Chem. 2011; 32:1456–1465. 21370243 10.1002/jcc.21759

[40] Dunning T.H. Gaussian basis sets for use in correlated molecular calculations. I. The atoms boron through neon and hydrogen. J. Chem. Phys. 1989; 90:1007–1023.

[41] Becke A.D. Density functional thermochemistry. III. The role of exact exchange. J. Chem. Phys. 1993; 98:5648–5652.

[42] Case D., Betz R., Cerutti D., Cheatham T.E., Darden T., Duke R., Giese T., Gohlke H., Goetz A., Homeyer N. et al. Amber 16. 2016; San Francisco University of California.

[43] Macke T., Case D. Leontes NB, SantaLucia J Jr. Modeling unusual nucleic acid structures. Molecular Modeling of Nucleic Acids. 1998; Washington, DC American Chemical Society 379–393.

[44] Bayly C.I., Cieplak P., Cornell W., Kollman P.A. A well-behaved electrostatic potential based method using charge restraints for deriving atomic charges: the RESP model. J. Phys. Chem. 1993; 97:10269–10280.

[45] Dupradeau F.-Y., Pigache A., Zaffran T., Savineau C., Lelong R., Grivel N., Lelong D., Rosanski W., Cieplak P. The R.E.D. tools: advances in RESP and ESP charge derivation and force field library building. Phys. Chem. Chem. Phys. 2010; 12:7821–7839. 20574571 10.1039/c0cp00111b PMC2918240

[46] Ivani I., Dans P.D., Noy A., Pérez A., Faustino I., Hospital A., Walther J., Andrio P., Goñi R., Balaceanu A. et al. Parmbsc1: a refined force field for DNA simulations. Nat. Methods. 2016; 13:55–58. 26569599 10.1038/nmeth.3658 PMC4700514

[47] Pérez A., Marchán I., Svozil D., Sponer J., Cheatham T.E., Laughton C.A., Orozco M. Refinement of the AMBER force field for nucleic acids: improving the description of α/γ conformers. Biophys. J. 2007; 92:3817–3829. 17351000 10.1529/biophysj.106.097782 PMC1868997

[48] Banáš P., Hollas D., Zgarbová M., Jurečka P., Orozco M., Cheatham T.E., Šponer J., Otyepka M. Performance of molecular mechanics force fields for RNA simulations: stability of UUCG and GNRA hairpins. J. Chem. Theory Comput. 2010; 6:3836–3849. 10.1021/ct100481h PMC8916691 35283696

[49] Zgarbová M., Otyepka M., Šponer J., Mládek A., Banáš P., Cheatham T.E., Jurečka P. Refinement of the Cornell et al. nucleic acids force field based on reference quantum chemical calculations of glycosidic torsion profiles. J. Chem. Theory Comput. 2011; 7:2886–2902. 21921995 10.1021/ct200162x PMC3171997

[50] Wang J., Wolf R.M., Caldwell J.W., Kollman P.A., Case D.A. Development and testing of a general amber force field. J. Comput. Chem. 2004; 25:1157–1174. 15116359 10.1002/jcc.20035

[51] Sousa da Silva A.W., Vranken W.F. ACPYPE - anteChamber PYthon parser interfacE. BMC Res. Notes. 2012; 5:367. 22824207 10.1186/1756-0500-5-367 PMC3461484

[52] Vögele M., Köfinger J., Hummer G. Molecular dynamics simulations of carbon nanotube porins in lipid bilayers. Faraday Discuss. 2018; 209:341–358. 29974904 10.1039/c8fd00011e

[53] Case D., Babin V., Berryman J., Betz R., Cai Q., Cerutti D., Cheatham T.E., Darden T., Duke R., Gohlke H. et al. Amber 14. 2014; San Francisco University of California.

[54] Jorgensen W.L., Chandrasekhar J., Madura J.D., Impey R.W., Klein M.L. Comparison of simple potential functions for simulating liquid water. J. Chem. Phys. 1983; 79:926–935.

[55] Joung I.S., Cheatham T.E. Determination of alkali and halide monovalent ion parameters for use in explicitly solvated biomolecular simulations. J. Phys. Chem. B. 2008; 112:9020–9041. 18593145 10.1021/jp8001614 PMC2652252

[56] Sindhikara D.J., Kim S., Voter A.F., Roitberg A.E. Bad seeds sprout perilous dynamics: stochastic thermostat induced trajectory synchronization in biomolecules. J. Chem. Theory Comput. 2009; 5:1624–1631. 26609854 10.1021/ct800573m

[57] Ryckaert J.-P., Ciccotti G., Berendsen H.J. Numerical integration of the cartesian equations of motion of a system with constraints: molecular dynamics of n-alkanes. J. Comput. Phys. 1977; 23:327–341.

[58] Berendsen H.J.C., Postma J.P.M., van Gunsteren W.F., DiNola A., Haak J.R. Molecular dynamics with coupling to an external bath. J. Chem. Phys. 1984; 81:3684–3690.

[59] Marko A., Margraf D., Yu H., Mu Y., Stock G., Prisner T. Molecular orientation studies by pulsed electron-electron double resonance experiments. J. Chem. Phys. 2009; 130:064102. 19222262 10.1063/1.3073040

[60] Lu X., Olson W.K. 3DNA: a software package for the analysis, rebuilding and visualization of three-dimensional nucleic acid structures. Nucleic Acids Res. 2003; 31:5108–5121. 12930962 10.1093/nar/gkg680 PMC212791

[61] Kumar R., Grubmüller H. dox3dna: a tool to analyze structural fluctuations of dsDNA or dsRNA from molecular dynamics simulations. Bioinformatics. 2015; 31:2583–2585. 25838463 10.1093/bioinformatics/btv190

[62] Humphrey W., Dalke A., Schulten K. VMD – visual molecular dynamics. J. Mol. Graphics. 1996; 14:33–38. 10.1016/0263-7855(96)00018-5 8744570

[63] Prisner T.F., Marko A., Sigurdsson S.T. Conformational dynamics of nucleic acid molecules studied by PELDOR spectroscopy with rigid spin labels. J. Magn. Reson. 2015; 252:187–198. 25701439 10.1016/j.jmr.2014.12.008

[64] Krepl M., Zgarbová M., Stadlbauer P., Otyepka M., Banáš P., Koča J., Cheatham T.E., Jurečka P., Šponer J. Reference simulations of noncanonical nucleic acids with different χ variants of the AMBER force field: quadruplex DNA, quadruplex RNA, and Z-DNA. J. Chem. Theory Comput. 2012; 8:2506–2520. 23197943 10.1021/ct300275s PMC3506181

[65] Zgarbová M., Luque F.J., Šponer J., Cheatham T.E., Otyepka M., Jurečka P. Toward improved description of DNA backbone: revisiting epsilon and zeta torsion force field parameters. J. Chem. Theory Comput. 2013; 9:2339–2354. 24058302 10.1021/ct400154j PMC3775469

[66] Zgarbová M., Šponer J., Otyepka M., Cheatham T.E., Galindo-Murillo R., Jurečka P. Refinement of the sugar-phosphate backbone torsion beta for AMBER force fields improves the description of Z- and B-DNA. J. Chem. Theory Comput. 2015; 11:5723–5736. 26588601 10.1021/acs.jctc.5b00716

