# Supplementary material for: High-resolution EPR distance measurements on RNA and DNA with the non-covalent Ǵ spin label
Source: Nucleic Acids Res. 2019 Nov 28;48(2):924–33. doi: 10.1093/nar/gkz1096 (PMC6954412; doi:10.1093/nar/gkz1096)
Supplement: gkz1096_Supplemental_File [file gkz1096_supplemental_file.pdf]

Supporting Information: High-resolution EPR distance measurements on RNA and DNA with the non-covalent Ǵ spin label

Marcel Heinz,[†,∥] Nicole Erlenbach,[‡,∥] Lukas S. Stelzl,[†] Grace Thierolf,[‡] Nilesh R. Kamble,[¶] Snorri Th. Sigurdsson,[¶] Thomas F. Prisner,[*,‡] and Gerhard Hummer[*,†,§]

†*Department of Theoretical Biophysics, Max Planck Institute of Biophysics, Max-von-Laue-Straße 3, 60438 Frankfurt am Main, Germany*

‡*Institute of Physical and Theoretical Chemistry and Center of Biomolecular Magnetic Resonance, Goethe University Frankfurt, Max-von-Laue-Straße 7, 60438 Frankfurt am Main, Germany*

¶*Department of Chemistry, Science Institute, University of Iceland, Dunhaga 3, 107 Reykjavík, Iceland*

§*Institute for Biophysics, Goethe University Frankfurt, 60438 Frankfurt am Main, Germany*

∥*Contributed equally to this work*

E-mail: prisner@chemie.uni-frankfurt.de; gerhard.hummer@biophys.mpg.de

Supporting Information

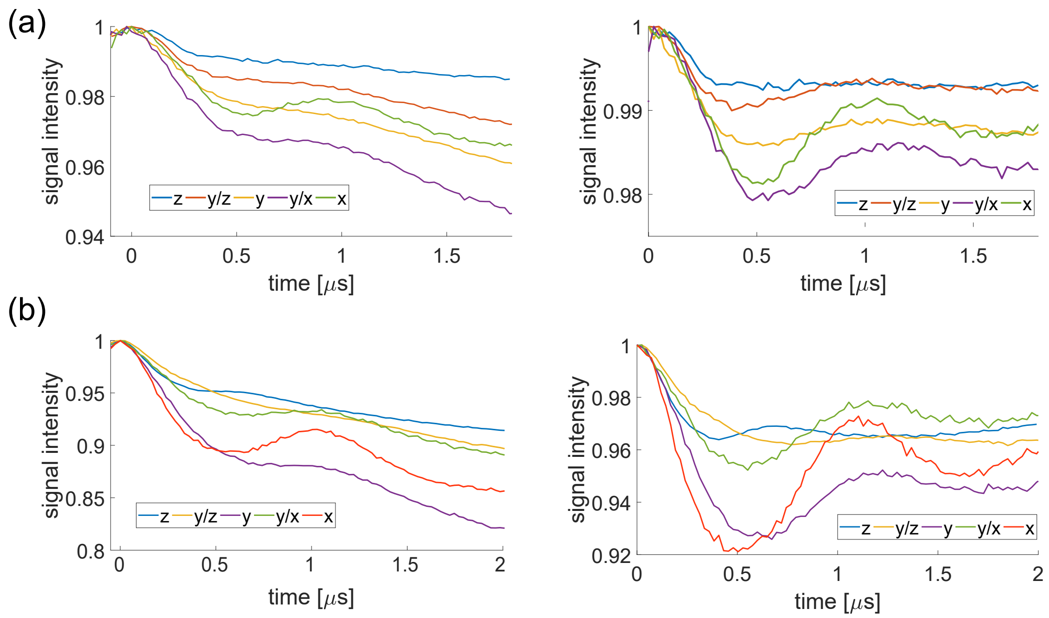

Supplementary Figure 1: G-band PELDOR time traces before (left) and after (right) background correction at the magnetic field positions corresponding to the main G-tensor components for (a) DNA (b) RNA.

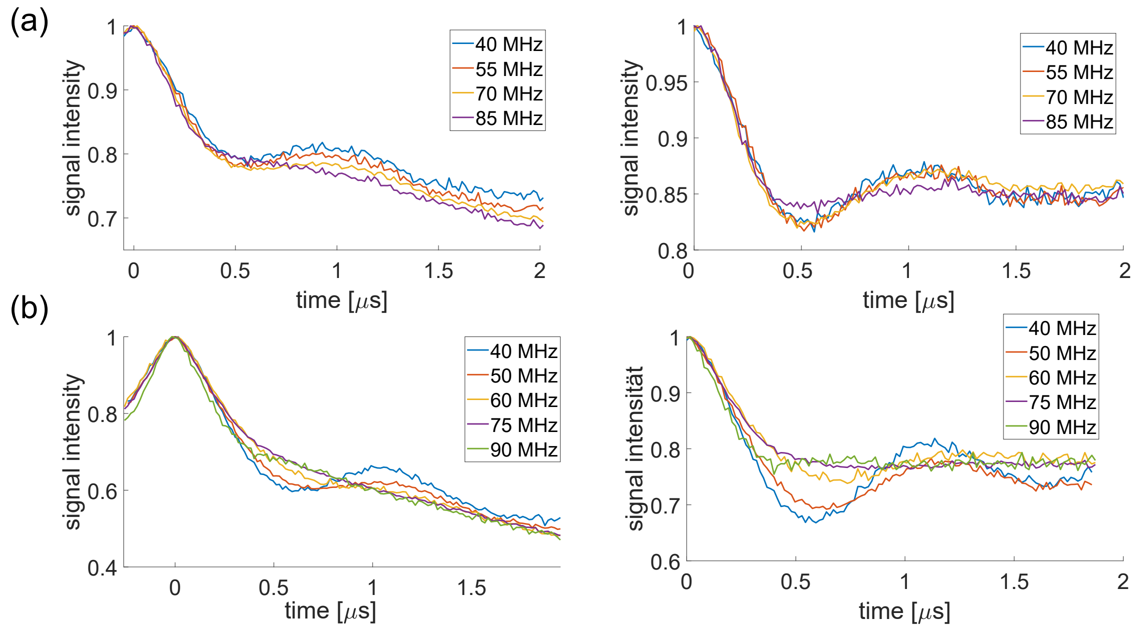

Supplementary Figure 2: X-band PELDOR time traces before (left) and after (right) background correction for (a) DNA and (b) RNA. Offset is indicated in the legend.

MD simulations

Minimization and equilibration

Energy minimizations were carried out in the sander program as implemented in Amber16.[1] Firstly, the solvent and ions were relaxed by 500 steps of steepest descent minimization and by 500 steps of conjugate gradient minimization. The nucleic acid atom positions and the **Ǵ** (G-spin) atom positions were restrained with a force constant of 500 kcal·mol^{-1}· Å^{-2} during this step. Non-bonded interactions were treated with a cut-off of 12 Å. Relaxations were performed with constant box volume. Secondly, the restraints were exclusively kept at the nucleic acid atoms, while relaxing the rest of the system. Thirdly, the whole system was free to relax with 1000 steps of steepest descent minimization and 1500 steps of conjugate gradient minimization.

The system was equilibrated in 50,000 steps with a time step of 2 fs ($\widehat{=}$ 100 ps). Weak atom position restraints (10 kcal·mol^{-1}· Å^{-2}) were applied at the atoms of the nucleic acid atoms. The solvent, ions, and **Ǵ** molecules were free to equilibrate. Random velocities were drawn from a Maxwell-Boltzmann distribution. Long-range electrostatic interactions were treated with the particle-mesh Ewald summation and a cut-off of 12 Å was applied for non-bonded real-space interactions. Covalent bonds to hydrogen atoms were constrained with the SHAKE algorithm. Langevin dynamics with a collision frequency of 1.0 ps^{-1} slowly heated the system up from 0 K to 300 K. In a second equilibration step, we switched from the sander to the pmemd.cuda engine in Amber16. The complete system was relaxed in 500 ps with a constant temperature of 300 K using Langevin dynamics (γ = 1.0 ps^{-1}). Isotropic position scaling with a relaxation time of 2 ps ensured an average pressure of 1 atm. A random seed was set at the restart. Non-bonded interactions were truncated after 12 Å and hydrogen atoms were constraint with SHAKE.

Atom types and partial charges of abasic sites and Ǵ spin label

Supplementary Table 1: Atoms, atom types, and partial charges (δ) of abasic site in RNA after the RESP fit. For comparison are the corresponding partial charges for a native guanosine shown, which are taken from the ParmBSC0+χ_{OL3} force field.

atom	abasic atom type	native residue δ [a.u.]	abasic residue δ [a.u.]
P	P	1.166 200	1.150 865
OP1	O2	−0.776 000	−0.790 585
OP2	O2	−0.776 000	−0.790 585
O5′	OS	−0.498 900	−0.404 835
C5′	CI	0.055 800	0.013 465
H5′	H1	0.067 900	0.060 165
H5″	H1	0.067 900	0.060 165
C4′	CT	0.106 500	0.128 465
H4′	H1	0.117 400	0.074 065
O4′	OS	−0.354 800	−0.465 635
C1′	CT	0.019 100	0.064 865
H1′	H1	0.200 600	0.038 965
H1″	H1	/	0.038 965
C3′	CT	0.202 200	0.067 265
H3′	H1	0.061 500	0.110 765
C2′	CT	0.067 000	0.307 565
H2′	H1	0.097 200	0.050 765
O2′	OH	−0.613 900	−0.748 535
HO2′	HO	0.418 600	0.459 365
O3′	OS	−0.524 600	−0.425 535
		$\sum\delta$=-0.8963	$\sum\delta$=-1.0000

Supplementary Table 2: Atoms, atom types, and partial charges (δ) of the abasic site in DNA after the RESP fit. For comparison are the corresponding partial charges for a native deoxyguanosine shown, which are taken from the ParmBSC1 force field.

atom	abasic atom type	native residue δ [a.u.]	abasic residue δ [a.u.]
P	P	1.165 900	1.084 847
OP1	O2	−0.776 100	−0.782 353
OP2	O2	−0.776 100	−0.782 353
O5′	OS	−0.495 400	−0.308 753
C5′	CI	−0.006 900	−0.118 353
H5′	H1	0.075 400	0.090 846
H5″	H1	0.075 400	0.090 846
C4′	CT	0.162 900	0.154 747
H4′	H1	0.117 600	0.060 947
O4′	OS	−0.369 100	−0.447 753
C1′	CT	0.035 800	0.010 547
H1′	H2	0.174 600	0.056 246
H1″	H2	/	0.056 246
C3′	CE	0.071 300	0.119 547
H3′	H1	0.098 500	0.096 447
C2′	CT	−0.085 400	−0.013 253
H2′	HC	0.071 800	0.031 847
H2″	HC	0.071 800	0.031 847
O3′	OS	−0.523 200	−0.432 153
		$\sum\delta$=-0.9112	$\sum\delta$=-1.0000

Supplementary Table 3: Atoms, GAFF atom types, partial charges (δ), x-,y- and z-coordinates of the **Ǵ** atoms.

atom	atom type	δ [a.u.]	coordinates x	y	z
N1	na	−0.348 400	−3.661	2.136	0.774
H1	hn	0.338 900	−2.946	2.773	1.041
C1	cc	0.250 800	−4.729	0.373	0.120
N2	nc	−0.564 100	−5.683	1.326	0.398
C2	cd	0.183 900	−5.019	2.348	0.781
H2	h5	0.144 900	−5.435	3.287	1.083
C3	cd	0.164 400	−3.474	0.865	0.350
N3	nd	−0.474 800	−2.275	0.251	0.203
C4	cc	0.548 400	−2.380	−0.963	−0.212
N4	n	−0.497 200	−3.575	−1.562	−0.479
H3	hn	0.336 200	−3.599	−2.494	−0.835
C5	c	0.484 200	−4.859	−0.978	−0.350
O1	o	−0.535 200	−5.821	−1.625	−0.628
N5	nh	−0.480 200	−1.290	−1.747	−0.427
H4	hn	0.328 300	−1.466	−2.717	−0.562
C6	ca	0.137 000	0.061	−1.417	−0.157
C7	ca	−0.275 900	0.610	−0.204	−0.546
H5	ha	0.202 500	−0.006	0.532	−1.022
C8	ca	0.011 000	1.949	0.031	−0.294
C9	ca	0.038 500	2.746	−0.925	0.313
C10	ca	−0.339 600	2.201	−2.140	0.685
H6	ha	0.203 900	2.801	−2.897	1.158
C11	ca	−0.084 200	0.857	−2.378	0.457
H7	ha	0.127 600	0.421	−3.313	0.762
C12	c3	0.196 200	2.734	1.283	−0.626
C13	c3	−0.272 100	2.233	2.518	0.130
H8	hc	0.082 400	2.169	2.320	1.194
H9	hc	0.082 400	1.249	2.807	−0.224
H10	hc	0.082 400	2.914	3.346	−0.027
C14	c3	−0.263 800	2.809	1.563	−2.130
H11	hc	0.080 900	3.150	0.687	−2.670
H12	hc	0.080 900	3.500	2.375	−2.319
H13	hc	0.080 900	1.833	1.843	−2.514
N6	n3	0.163 000	4.060	0.892	−0.123
O2	o	−0.398 500	5.043	1.662	−0.197
C15	c3	0.257 600	4.173	−0.448	0.475
C16	c3	−0.237 200	4.603	−0.329	1.941
H14	hc	0.068 200	4.677	−1.311	2.397
H15	hc	0.068 200	3.887	0.256	2.507
H16	hc	0.068 200	5.570	0.154	2.004
C17	c3	−0.258 400	5.181	−1.288	−0.316
H17	hc	0.072 600	6.157	−0.819	−0.285
H18	hc	0.072 600	4.876	−1.384	−1.351
H19	hc	0.072 600	5.264	−2.283	0.110

Collective variable describing Ǵ conformations

The dihedral angle **d1** describes the accessible **Ǵ** conformations in a dsNA helix. It has been shown that a TEMPO spin label covalently attached at a cytosine base[2] could be characterized via rotations of two torsion angles Φ_1 and Φ_2. Following this approach, the potential energy surface (PES) for the **Ǵ** was constructed, based on the torsion angles Φ_1 and Φ_2 (SI Figure 3). Six minima were found, where Φ_1 adopts 180° (minima 1, 2, 5, and 6) or 0° (minima 3, and 4). Φ_2 spans a larger variety of angles, i.e., 130° (1), −50° (2), 180° (3), 0° (4), 40° (5), −140° (6). Larger energy barriers (9 kcal/mol) separate the minima 1, 2, 5, and 6 from minima 3, and 4. In context of a **Ǵ** molecule incorporated in a dsNA, Watson-Crick hydrogen bond formation needs to be accessible, revealing that minima 1, 2, 5, and 6 will interfere the hydrogen bond formation and can be neglected. Accessible Watson-Crick edges are only present for minima 3 and 4. The comparison between relaxed surface scan around **d1** (SI Figure 3, green dots) and the present PES reveal that the single collective variable **d1** is sufficient to trace the local minimum-energy pathway near Φ_1=0 of the possible **Ǵ** conformations in a dsNA structure.

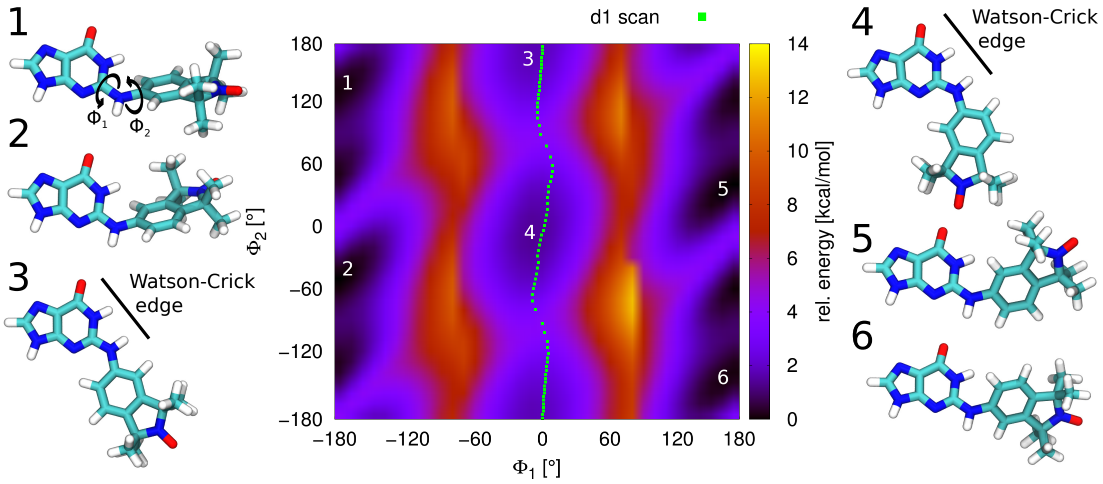

Supplementary Figure 3: Potential energy surface of a **Ǵ** molecule in dependence of torsion angles Φ_1 and Φ_2, constructed at a PBE0/N07D level of theory (step size 10°). Minima and their representative structures are shown (1–6). Watson-Crick edges are only accessible for minima 3 and 4. Green dots represent the corresponding structures of the relaxed surface scan of dihedral angle **d1**, as discussed in the main text.

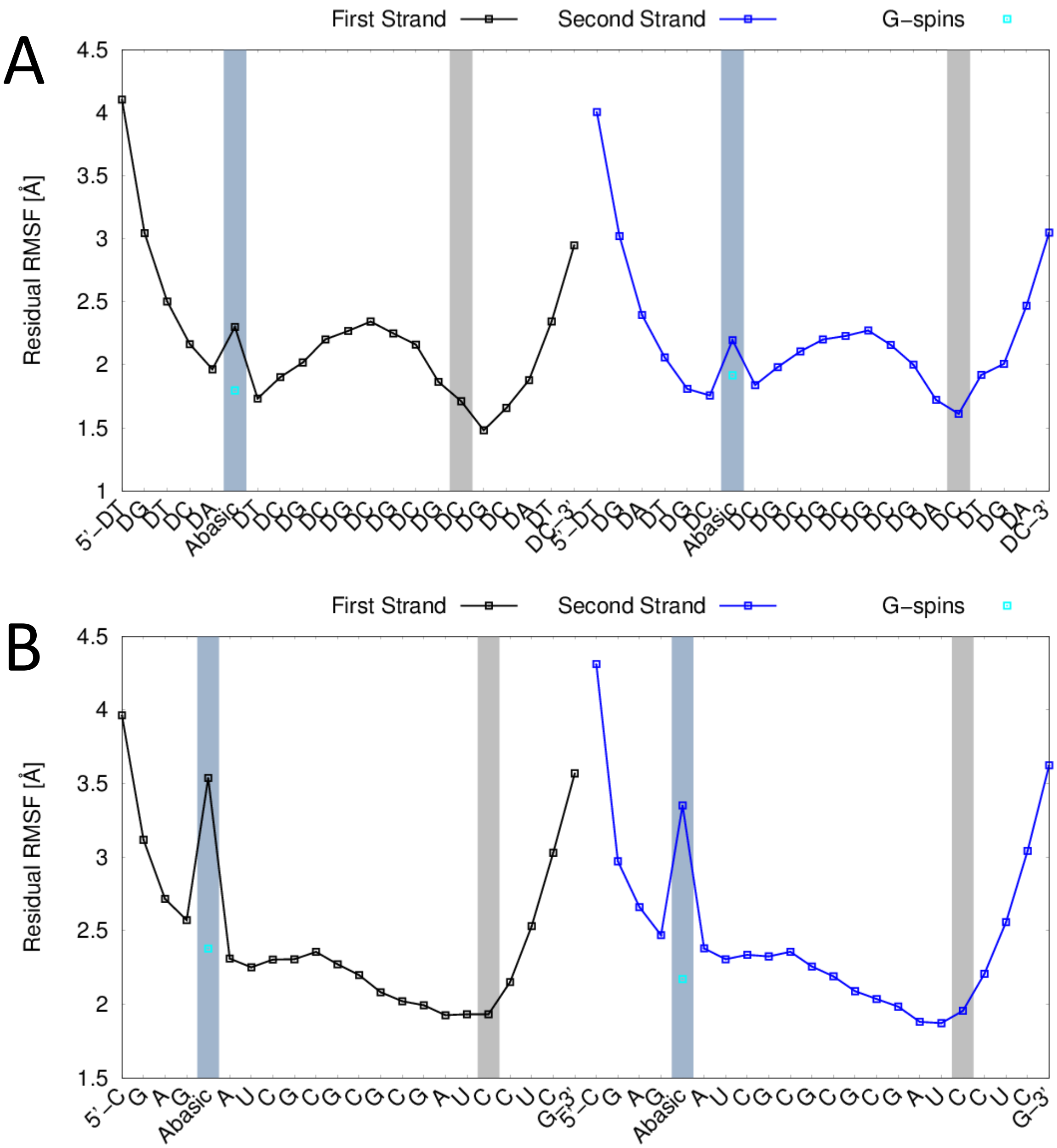

Supplementary Figure 4: Root mean square fluctuations for each residue in labeled DNA (A) and labeled RNA (B). The averaged structures of each trajectory served as the references. Abasic sites are highlighted in gray and the corresponding residue in the complementary strand in light-gray.

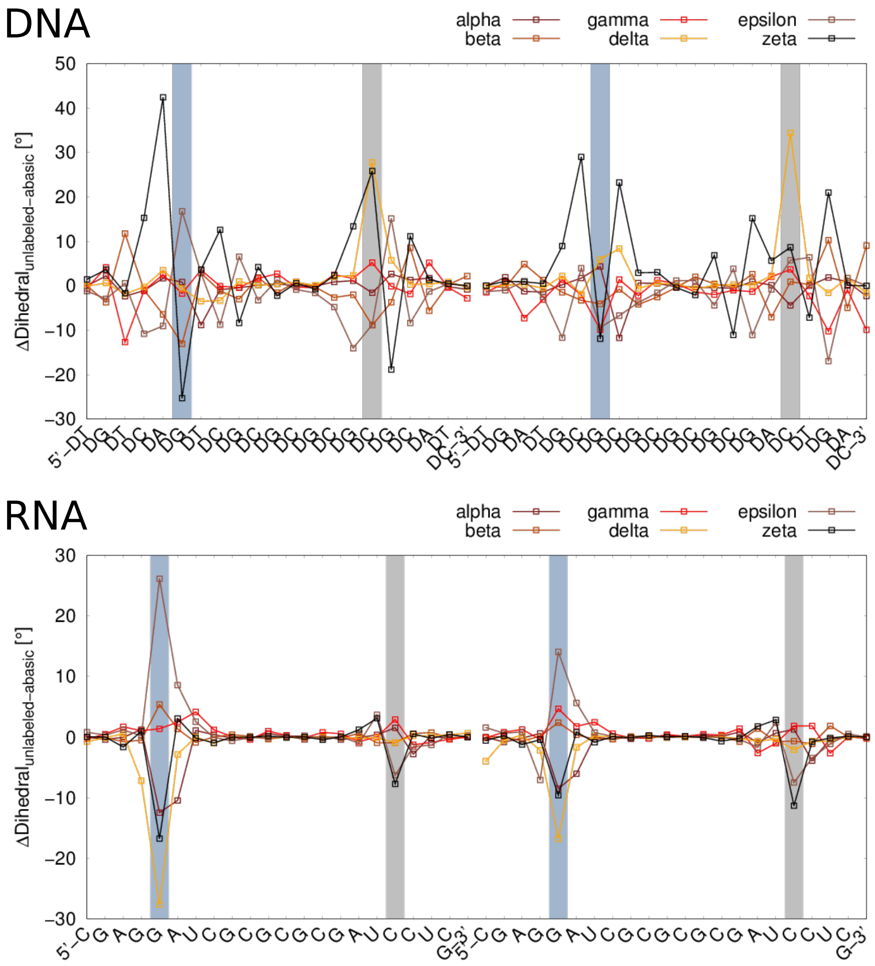

Supplementary Figure 5: Change in backbone dihedral angles of dsDNA (top) and dsRNA (bottom) induced by **Ǵ**. The difference (unlabeled-labeled) of the average backbone dihedral angles in unlabeled and labeled DNA is shown. Abasic sites are highlighted in gray and the corresponding residue in the complementary strand in light-gray.

Umbrella sampling

Umbrella sampling simulations were performed in Amber16.[1] PMF profiles were obtained using the Weighted Histogram Analysis Method (WHAM).[3]

The isolated **Ǵ** (in vacuo) was restrained with a small harmonic force constant (k = 50 kcal·mol^{-1}·rad^{-2}) at Φ_1≈0° to maintain conformations amenable to Watson-Crick base pairing, while sampling **d1**. The umbrella windows for **d1** were spaced 5° apart from each other, ranging from −180° to 180°. The force constant was set to 200 kcal·mol^{-1}·rad^{-2}. As an initial structure, **d1** was constrained to 0°. The structure was minimized in 500 steps with the steepest descent algorithm followed by 1500 steps with conjugate gradient algorithm. The non-bonded cut-off was set to 999 Å. The resulting structure was then equilibrated in 500 ps (Δt = 1 fs). The system was slowly heated up from 0 K to 300 K using Langevin dynamics with a collision frequency of 1.0 ps^{-1}. A production run was performed for 100 ns.

The **Ǵ** structure with Φ_1=**d1**=0° was used to generate starting structures for **d1** = 60° and −60°, which where the used as starting structures for **d1** = 120° and −120°, and the structure with **d1** = 120° for a structure with **d1** = 180°. These structures were then taken as starting structures for the various windows, which were simulated as described for **d1**=0°.

The initial structures of **Ǵ** molecules inside dsDNA were obtained by using the structures after 1 μs of the MD simulations of the complete system. The dsDNA system contained two **Ǵ** molecules, where both **d1** were set to 120° and, in a separate simulation set up, to 180° with a force constant of 200 kcal·mol^{-1}·rad^{-2}. Systems were minimized in 500 steps with steepest descent algorithm followed by 1500 steps with conjugate gradient algorithm under constant box volume. The non-bonded cut-off was set to 12 Å. Systems were equilibrated for 500 ps, with a time step of 1 fs. The temperature was slowly increased from 0 K to 300 K using the Langevin dynamics with a collision frequency of 1 ps^{-1}. Hydrogen containing bonds were constrained using SHAKE. Average pressure of

1 atm was maintained by isotropic position scaling with a relaxation time of 5 ps. Non-bonded interactions were truncated at 12 Å. Production runs were performed for 1 ns. The resulting structures were taken to rotate **d1** for the next ± 60° structures, following the same protocol, as previously. Structures for 0°, ± 60°, ± 120° and 180° were generated, which were used to spawn of structures every 5°, ranging from 180° to −180°. With the starting structures every 5° (73 structures) on hand, longer production runs in ten blocks, each 4 ns (=40 ns), for every **d1** value, were performed (SI Figure 6), after a minimization and equilibration. The **d1** values were saved every 50 fs.

Structures for **Ǵ** molecules inside dsRNA were taken after 1 μs of MD simulations. The 1st **Ǵ** had a **d1** value close to 0°, while the 2nd **Ǵ** had a **d1** value close to 180°. The 2nd **Ǵ** was rotated to **d1** = 120°, afterwards to 60° and finally to 0°, following the described protocol as in dsDNA. With both **Ǵ** molecules inside dsRNA having **d1** =0°, the previously described protocol for dsDNA was repeated for dsRNA.

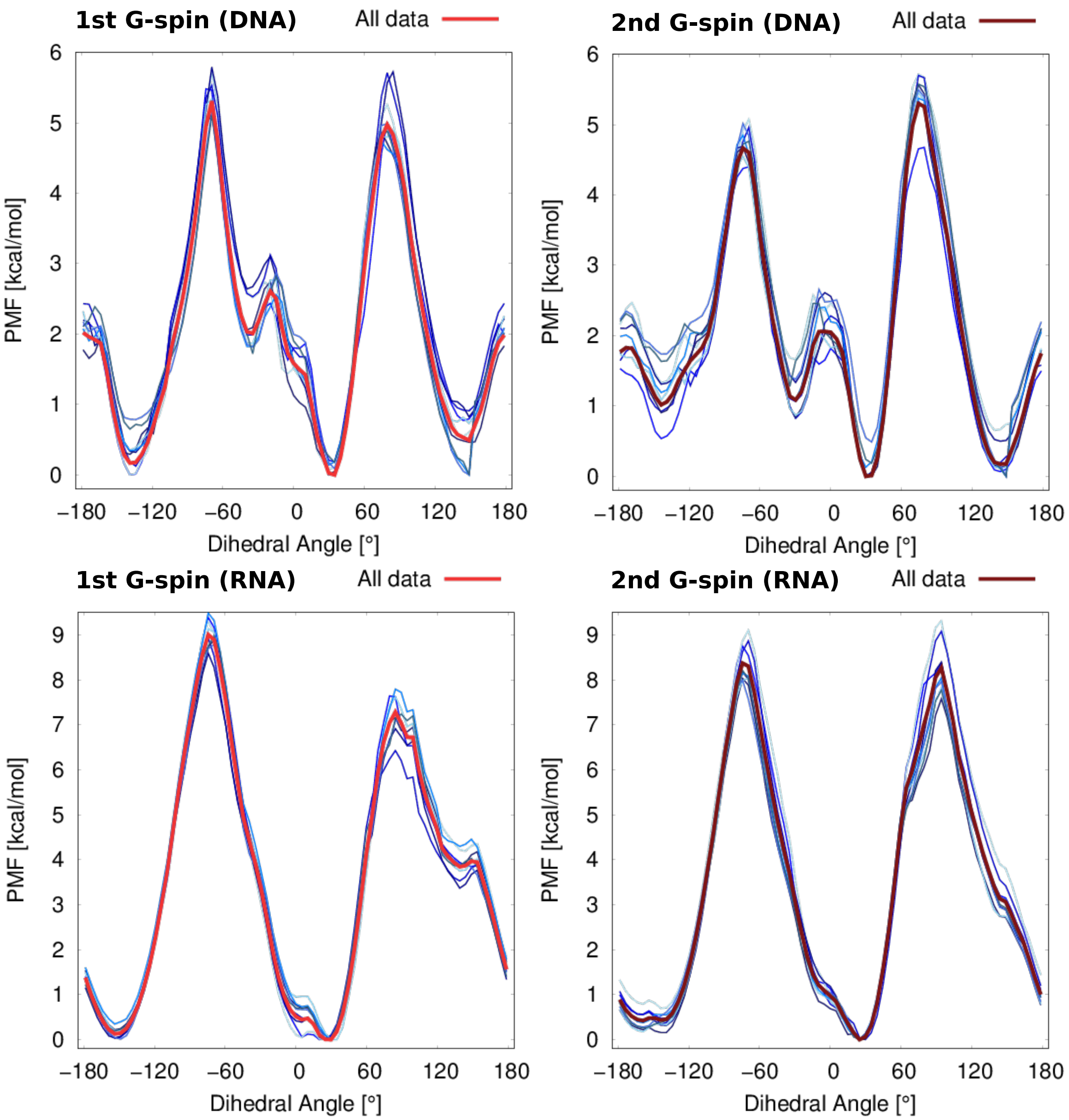

Supplementary Figure 6: PMF profiles of **Ǵ** molecule rotations around dihedral **d1** inside double-stranded nucleic acids. Ten blocks of 4 ns each were analyzed individually (blue lines). Red curves represent the PMF profile constructed out of all blocks.

Evaluation: Classical description of d1 rotation in Ǵ

We were interested if the force field employed for the **Ǵ** molecule is able to capture the main characteristics of the collective variable **d1**. The overall shape of the potential energy surface at a PBE0/N07D level of theory, describing a rotation around **d1**, was used as a reference (SI Figure 7). The global minimum was at $0°$ and an additional local minimum was found at **d1**=$180°$. Both minima were separated by barriers of $\approx$2.5 kcal/mol at $75°$ and $-75°$.

The free energy profile of the rotation around dihedral angle **d1** in the classically described **Ǵ** (vacuo) revealed a somewhat similar shape, with small deviations (SI Figure 7). Two minima at $33°$ and $-33°$ were separated by a small energy barrier of ~0.5 kcal/mol ($0°$). These two minima correspond to the minimum in the DFT calculation at $0°$ (syn). Due to the very small energy barrier between the classical minima, and the fact, that the DFT-calculated energies at this region are below 1 kcal/mol, we approximate them to a single minimum (syn), as mentioned in the main text. Additionally, two classically described minima at $133°$ and at $-133°$ were separated by a barrier of ~0.3 kcal/mol, indicating almost no barrier between the two minima. As these two minima correspond to the calculated minima ($180°$) at a DFT level of theory, we also approximate these two minima to a single minimum (anti). The syn and anti minima were separated by barriers of $\approx$2 kcal/mol at $75°$ and $-75°$, in good agreement with the DFT calculation (~2.5 kcal/mol).

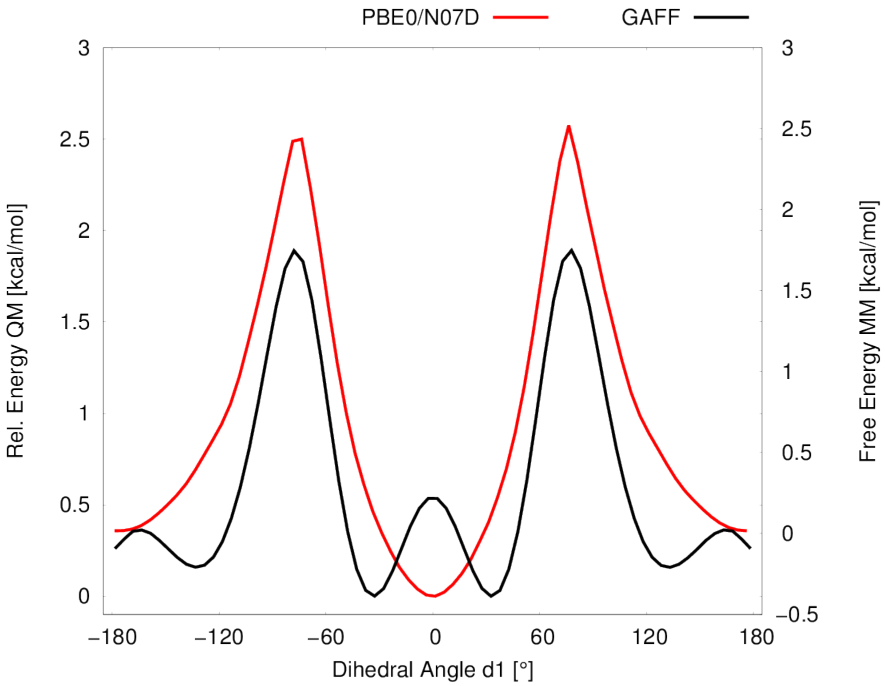

Supplementary Figure 7: Potential energy surface of a rotation around dihedral **d1** of **Ǵ** at a PBE0/N07D level of theory (red) and the free energy profile of the **Ǵ** molecule in vacuo classically described with GAFF (black).

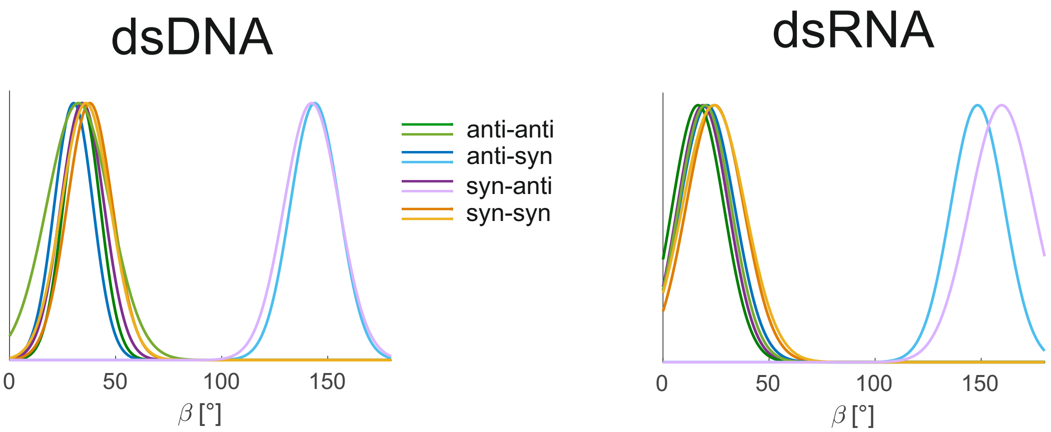

Supplementary Figure 8: Beta angle distribution for **Ǵ** labeled dsDNA and dsRNA extracted from MD simulations.

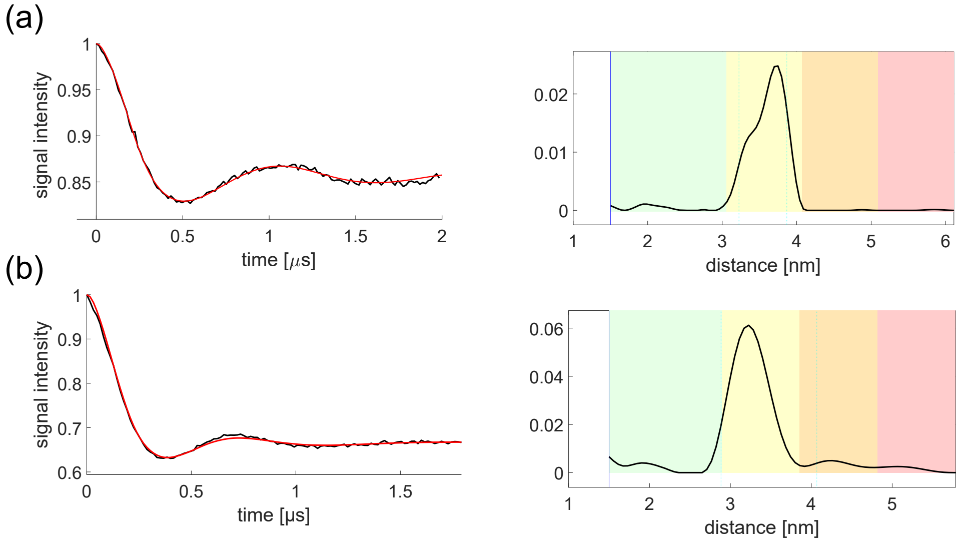

Supplementary Figure 9: (left) Summed PELDOR time traces (black) with the Tikhonov fit (red) for dsDNA (a) and dsRNA (b). (right) The corresponding distance distributions. Distances were derived with DeerAnalysis.[4] Background color coding indicates the confidence, with decreasing confidence from green (<3 nm) to red (>5 nm, where distances cannot be resolved).

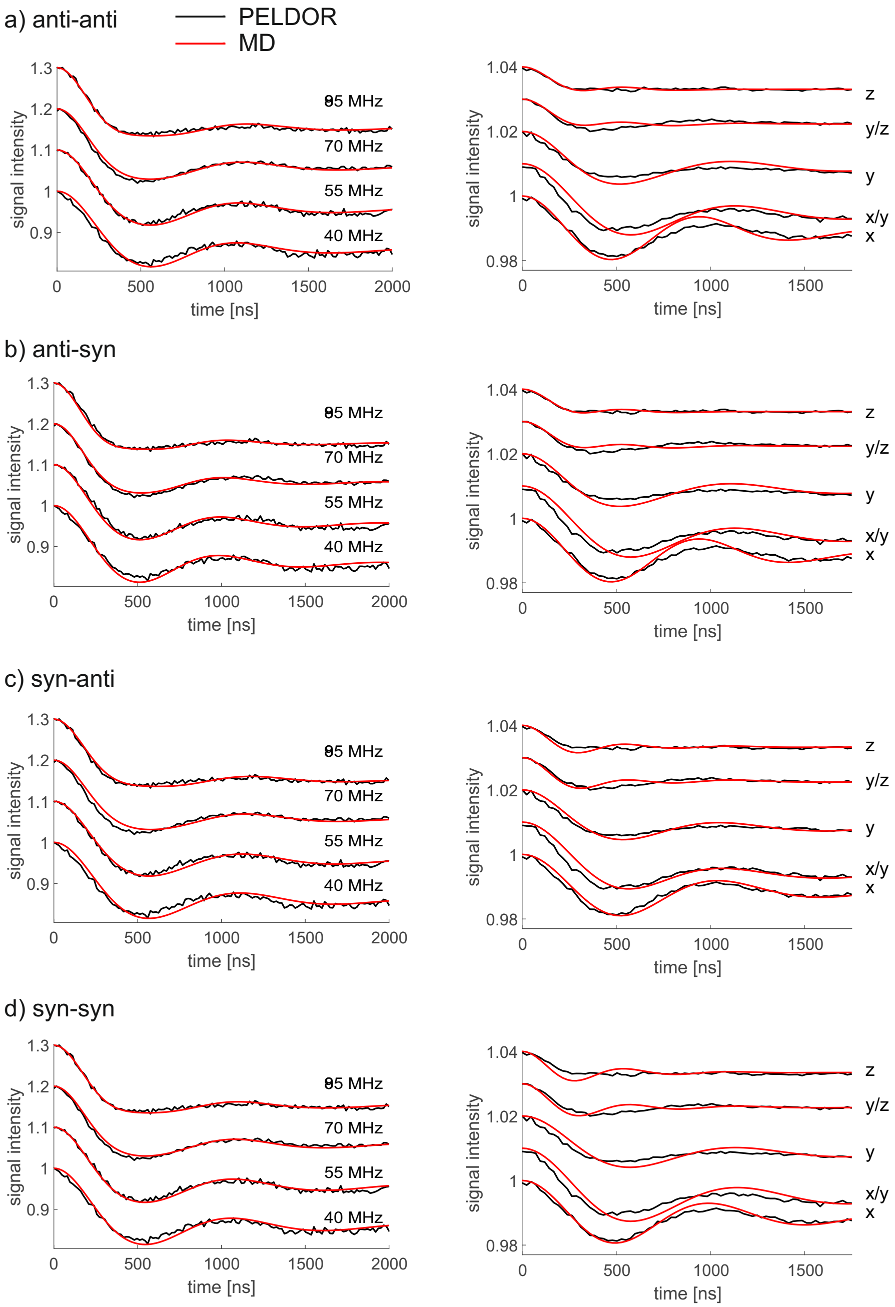

Supplementary Figure 10: Individual PELDOR time traces extracted from MD simulations (red) for different conformational states of **Ǵ** in dsDNA. Black lines show the experimental PELDOR time traces, not resolving individual states.

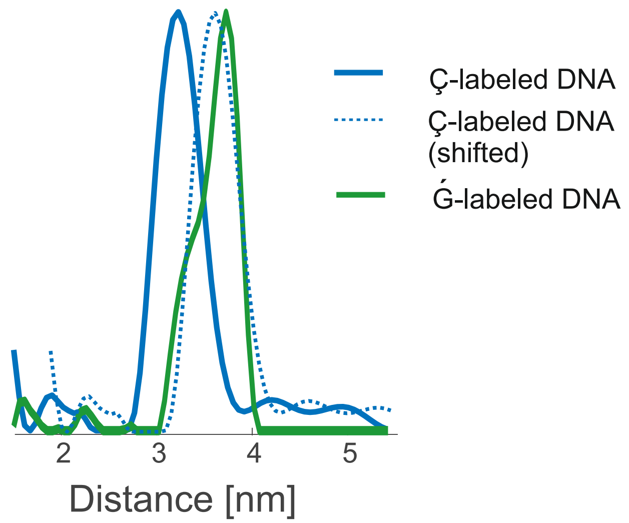

Supplementary Figure 11: Comparison of the distance distributions between **Ç** labeled DNA and **Ǵ** labeled DNA. The **Ç** labeled distance distribution is taken from Stelzl *et al.*[5] and shifted (dotted line) towards the distance distribution of **Ǵ** labeled DNA.

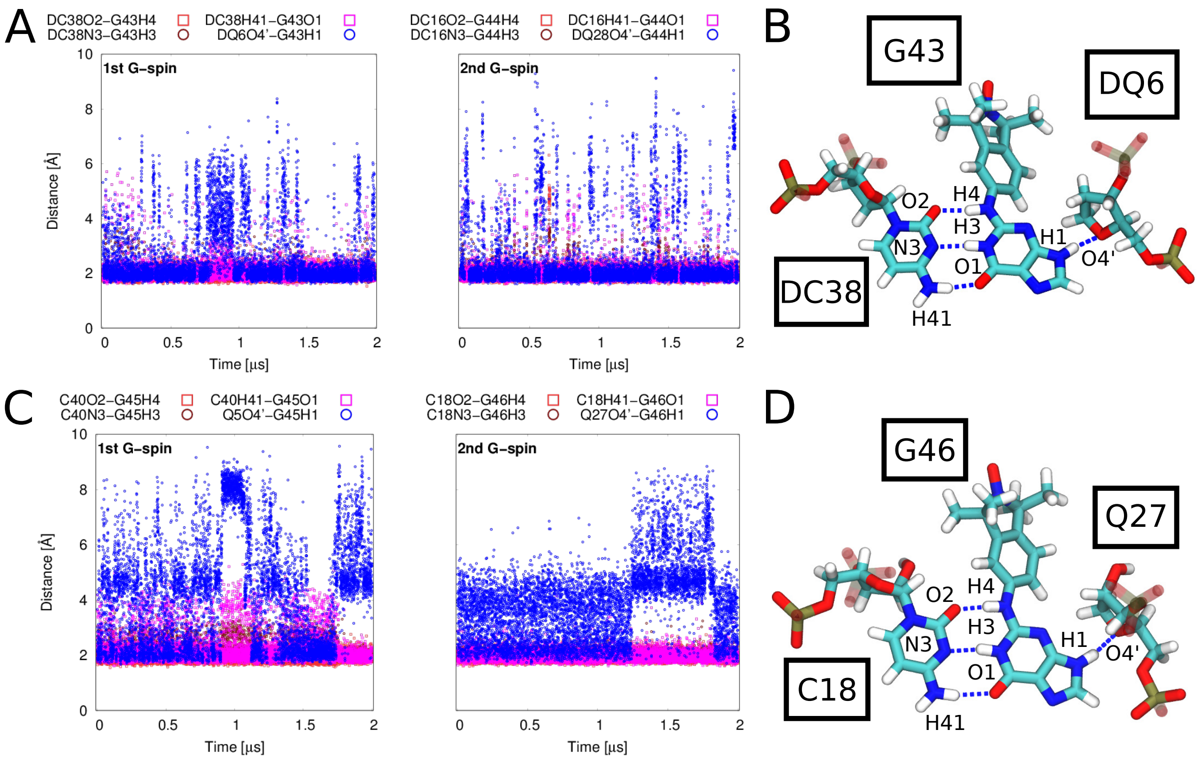

Supplementary Figure 12: Hydrogen bonds between two **Ǵ** molecules and their complementary cytidines and the abasic sites in dsDNA and dsRNA. (**A**)The three stable Watson-Crick H-bonds in dsDNA are shown with their distances as a function of time during the simulations (1st **Ǵ**: DC38O2-G43H4, DC38H41-G43O1, DC38N3-G43H3; 2nd **Ǵ**: DC16O2-G44H4, DC16H41-G44O1, DC16N3-G44H3). Additional hydrogen bonds between **Ǵ** molecules and the O4′ of the abasic sites are depicted in blue and remain stable within the simulations (1st **Ǵ**: DQ6O4′-G43H1; 2nd **Ǵ**: DQ28O4′-G44H1). (**B**) H-bonds between **Ǵ** (G43), the abasic site (DQ6) and the cytidine (DC38) are depicted as dashed lines. (**C**) The three stable Watson-Crick H-bonds in dsRNA are shown with their distances during the simulations (1st **Ǵ**: C40O2-G45H4, C40H41-G45O1, C40N3-G45H3; 2nd **Ǵ**: C18O2-G46H4, C18H41-G46O1, C18N3-G46H3). Additional hydrogen bonds between **Ǵ** molecules and the O4′ of the abasic sites are depicted in blue and remain stable within the simulations (1st **Ǵ**: Q5O4′-G45H1; 2nd **Ǵ**: Q27O4′-G46H1). (**D**) H-bonds between **Ǵ** (G46), the abasic site (Q27), and the cytidine (C18) are depicted as dashed lines.

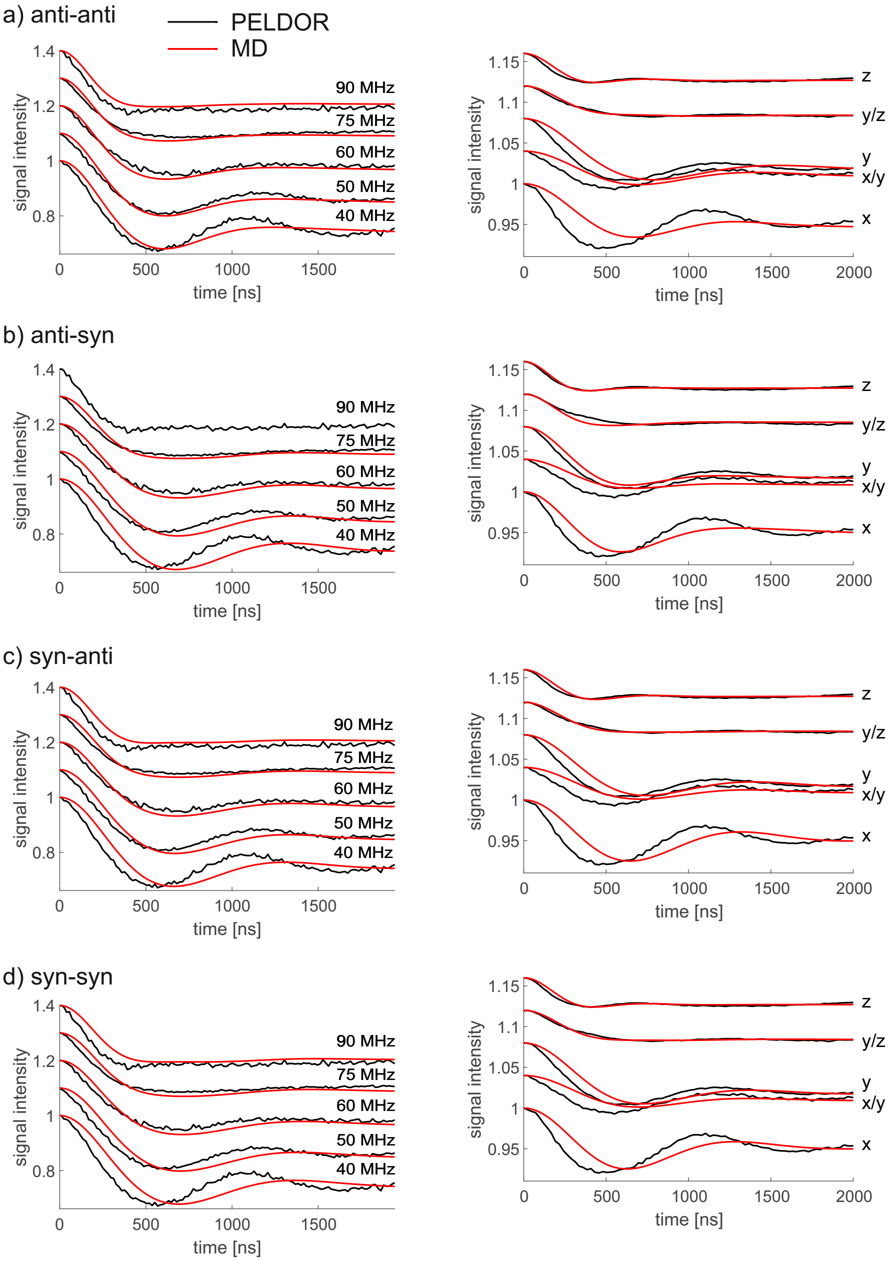

Supplementary Figure 13: Individual PELDOR time traces extracted from MD simulations for different conformational states of Ǵ in dsRNA. Black lines reflect the experimental PELDOR time traces, not resolving individual states.

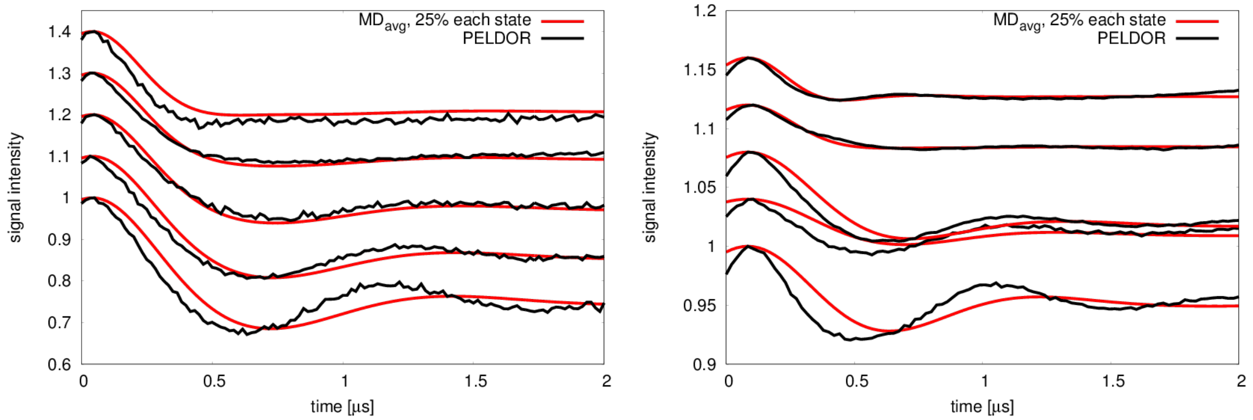

Supplementary Figure 14: Averaged PELDOR time traces over the four different **Ǵ** conformational states in dsRNA with equal weights (red). The X-band data (left) and G-band date (right) are the averages over the averaged PELDOR time traces for every state (SI Figure 13). The experimental PELDOR time traces are shown for clarity (black).

Average helix parameter

Helix and base pair parameters were calculated with the do_x3dna program package. The average parameter for twist, roll, slide, inclination, h-rise, and propeller were directly calculated from do_x3dna. The distributions of the values are peaked and the average values for χ, α, β, γ, δ, ϵ and ζ angles were calculated according to the mean of circular quantities for every frame (Eq. 1)

$$
\begin{aligned}
a &= \text{atan2}\left(\frac{1}{n}\cdot\sum_{j=1}^{n}\sin\theta_j\,,\ \frac{1}{n}\cdot\sum_{j=1}^{n}\cos\theta_j\right)\\
\bar{\theta} &= \begin{cases} a & a \geq 0 \\ a + 360^\circ & a < 0 \end{cases}
\end{aligned}
\tag{1}
$$

where $\bar{\theta}$ is the average angle over n θ angles. The individual base pair averages were then averaged over the whole sequence (SI Table 4). The errors were calculated as the standard deviation of the base pair averages to the overall average, divided by the number of base pairs ($\sigma/\sqrt{N}$).

Supplementary Table 4: The representative mean structural parameter of the inner base pairs (AUCGCGCGCGAU), flanked by the two **Ǵ** positions in dsRNA.

parameter	labeled	unlabeled
twist [°]	30.5 ± 1.3	30.0 ± 1.2
roll [°]	6.9 ± 1.9	6.8 ± 1.9
slide [Å]	-1.7 ± 0.2	-1.7 ± 0.1
inclination [°]	12.2 ± 3.4	12.2 ± 3.4
h-rise [Å]	2.8 ± 0.2	2.8 ± 0.2
propeller [°]	347.4± 2.5	348.4 ± 2.5
χ [°]	199.3 ± 0.6	199.8 ± 0.6
α [°]	284.2 ± 0.3	284.2 ± 0.3
β [°]	173.3 ± 0.3	172.8 ± 0.2
γ [°]	64.3 ± 0.3	64.8 ± 0.1
δ [°]	78.9 ± 0.3	78.6 ± 0.2
ϵ [°]	202.5 ± 0.7	201.9 ± 0.4
ζ [°]	291.9 ± 0.3	291.7 ± 0.3

References

(1) Case, D. et al. AMBER 16. 2016; University of California, San Francisco.

(2) Halbmair, K.; Seikowski, J.; Tkach, I.; Höbartner, C.; Sezer, D.; Bennati, M. *Chem. Sci.* **2016**, *7*, 3172–3180.

(3) Grossfield, A. 'WHAM: the weighted histogram analysis method", version 2.0.9. `http://membrane.urmc.rochester.edu/wordpress/?page_id=126`.

(4) Jeschke, G.; Chechik, V.; Ionita, P.; Godt, A.; Zimmermann, H.; Banham, J.; Timmel, C. R.; Hilger, D.; Jung, H. *Appl. Magn. Reson.* **2006**, *30*, 473–498.

(5) Stelzl, L. S.; Erlenbach, N.; Heinz, M.; Prisner, T. F.; Hummer, G. *J. Am. Chem. Soc.* **2017**, *139*, 11674–11677.
